# Design and analysis of randomized clinical trials for onchocerciasis, loiasis and mansonellosis: A systematic review

**DOI:** 10.1371/journal.pntd.0013992

**Published:** 2026-02-20

**Authors:** Fabrice Lotola Mougeni, Marta Bofill Roig, Marc P. Hübner, Ute Klarmann-Schulz, Benjamin Lenz, Sabine Specht, Martin Posch, Sonja Zehetmayer

**Affiliations:** 1 Center for Medical Data Science, Medical University of Vienna, Vienna, Austria; 2 Department of Statistics and Operations Research and Institute for Research and Innovation in Health (IRIS), Universitat Politècnica de Catalunya - BarcelonaTech (UPC), Barcelona, Spain; 3 University of Bonn, University Hospital Bonn, Institute for Medical Microbiology, Immunology and Parasitology, Bonn, Germany; 4 German Center for Infection Research (DZIF), Partner Site Bonn-Cologne, Bonn, Germany; 5 Filarial Disease, Drugs for Neglected Diseases initiative, Geneva, Switzerland; Universiteit Antwerpen, BELGIUM

## Abstract

**Background:** The design and analysis of randomized clinical trials (RCTs) in filarial diseases such as onchocerciasis, loiasis, and mansonellosis pose unique statistical challenges, including skewed endpoints and limited sample sizes. This systematic review summarizes design and analysis approaches of RCTs conducted in these diseases with a focus on the statistical methodology.

**Methods and findings:** A systematic search was conducted in PubMed and four trial registries to identify RCTs investigating treatments for onchocerciasis, loiasis, and mansonellosis published or registered between 2000 and 2024. We excluded studies focusing on new methods or pharmacokinetics, short reports, and Phase I trials. Forty-four studies met the inclusion/exclusion criteria (23 for onchocerciasis, 16 for loiasis, and 5 for mansonellosis), information was retrieved from the registries, the manuscripts and/or the study protocol. As primary efficacy endpoints, for onchocerciasis studies qualitative endpoints dominated, while quantitative endpoints were more frequently observed for loiasis and mansonellosis. The most frequently reported hypothesis tests for the primary endpoint were the Mann-Whitney U and the chi-squared tests. We found considerable heterogeneity between trials - not only in study-specific parameters such as the number of arms, type of blinding or control group - but also in design parameters or attributes that could be standardized within each disease across studies with similar objectives, such as the primary endpoint, length of follow-up, the analysis method and the primary analysis population.

**Conclusions:** Several trials were well-planned with detailed information provided in either the manuscript or the registry. However, for some trials, information was sparse or incomplete, indicating a need for more structured and transparent reporting. Adopting established frameworks such as CONSORT and ICH E9 (R1) estimand approach would enhance transparency and better align trial objectives, analyses, and reported conclusions.

## Author summary

Filarial diseases such as onchocerciasis (“river blindness”), loiasis (“African eye worm”), and mansonellosis affect millions of people, mainly in tropical regions. Developing effective treatments for these diseases requires carefully designed clinical trials. However, conducting and analyzing these trials can be difficult because patient numbers are often small and the measurements used to evaluate treatment success can vary widely. In this study, we systematically reviewed randomized clinical trials from the past 25 years that tested treatments for these filarial diseases. We examined the design of these trials, the analysis of the results, and the statistical methods applied. We found that, although many studies were well planned, there was substantial variation in key trial characteristics, including primary outcome measures, duration of follow-up, and data analysis methods. In several trials, essential details were insufficiently reported, which limited comparisons across studies. Clearer and more consistent reporting together with the use of advanced statistical methods, would make research on these diseases more reliable and easier to interpret.

## Introduction

Filarial diseases such as onchocerciasis, loiasis, and mansonellosis pose a substantial health burden in many low-resource settings, particularly in Sub-Saharan Africa [1–9]. In particular, onchocerciasis is associated with substantial morbidity and economic hardship in endemic regions, while the contribution of loiasis and particularly mansonellosis has been less well investigated [10].

Onchocerciasis, also known as river blindness, is caused by the filarial round worm *Onchocerca volvulus*, and is one of the most extensively studied neglected tropical diseases (NTDs) due to its severe impact on affected communities [11]. The disease affects approximately 21 million people in Sub-Saharan Africa and can lead to vision loss, blindness and severe dermatitis [9,12]. Treatments such as doxycycline (DOXY), an individual macrofilaricidal (adult worm killing) therapy, or ivermectin (IVM), a microfilaricidal, transmission inhibiting treatment used for mass drug administration (MDA), are widely used [13].

Loiasis is caused by the filarial nematode *Loa loa*, commonly known as the African eye worm, and is endemic in central and western Sub-Saharan Africa with an estimated 20 million infected [14]. Clinically, loiasis is mainly known for the migration of the adult worms through the eye, the subcutaneous tissues and the development of Calabar swellings. More recently, loiasis has been associated with increased mortality and atypical chronic symptoms [15]. Most importantly, individuals with a high burden of filarial progeny, the microfilariae (mf), in peripheral blood, are at risk of life-threatening adverse events following treatment with drugs that kill the mf. Thus, loiasis-endemic areas are often excluded from MDA programs (test and not treat approach). For individual treatment with IVM or diethylcarbamazine (DEC), participants have to be checked for their mf counts before treatment is initiated, which is usually not done during MDA campaigns [16].

Mansonellosis caused by *Mansonella perstans*, *Mansonella ozzardi*, and *Mansonella streptocerca* is estimated to affect over 120 million individuals primarily in rural regions of Central Africa, the Caribbean, and South America. For *M. perstans*, adult worms typically live in body cavities, while their larvae (mf), circulate in the blood. Among available treatments, DOXY has been shown to significantly and sustainably reduce mf levels [17,18]. In contrast to onchocerciasis and loiasis, mansonellosis is thought to be an infection that is often asymptomatic or associated with unspecific symptoms [19]. Moreover, immunomodulation of the filariae may increase the risk of co-infections such as HIV and tuberculosis, and may reduce immune responses to vaccines [5,20,21]. Single treatments with microfilaricidal drugs used for MDA of onchocerciasis or loiasis have limited impact on *M. perstans* mf numbers [22,23].

Randomized clinical trials (RCTs) are the gold standard for evaluating the efficacy and safety of novel treatments. Due to the difficulty of directly measuring the abundance of adult worms, RCTs in onchocerciasis, loiasis, and mansonellosis often use mf as primary or secondary endpoints. Another challenge is the inconsistency of methods used to detect and quantify mf, which can substantially affect the sensitivity and counts obtained. Endpoints are also defined either qualitatively or quantitatively. Qualitative endpoints typically involve binary outcomes, such as achieving a 50% reduction in mf at follow-up compared to baseline or complete clearance of mf [24,25]. Quantitative endpoints include mf counts (e.g., mf per mg of skin or per ml of blood) or a continuous measure of change in mf from baseline to follow-up [26,27]. There is currently no consensus on the optimal timing of follow-up or on endpoint definitions for the three considered diseases. The choice of endpoint and follow-up duration may also depend on the mode of action of the investigated treatments. Heterogeneity in trial inclusion criteria (e.g., mf counts above a predefined level), trial characteristics such as population-wide versus targeted treatment, baseline infection prevalence, or history of previous MDAs complicate comparison of clinical trials, as they affect the estimated drug efficacy as well as the rate of new infections.

The design and analysis of RCTs in these filarial diseases pose specific statistical challenges. Mf count data are typically right-skewed and therefore not normally distributed. Transformations, such as log transformation, can be used to approximate normality, enabling group comparisons with parametric tests such as t-test or analysis of variance. Alternatively, nonparametric methods, such as the Mann-Whitney U or Kruskal-Wallis tests, are often used to compare skewed distributions between treatment groups. Alexander [28] reviewed statistical methods for analyzing skewed parasite count data and discussed their advantages and disadvantages. The article emphasized the lack of clarity in reporting measures of central tendency and supported the use of either arithmetic or geometric means depending on the scientific context. The authors also raised their concern regarding the inappropriate use of parametric tests, such as the t-test, and recommended non-parametric approaches or generalized linear models. However, the review did not specifically address applications in RCTs for filarial diseases.

The eWHORM study (EU Grant agreement ID: 101103053, https://ewhorm.org/) aims to conduct an adaptive randomized controlled clinical basket trial to evaluate the efficacy and safety of the drug oxfendazole compared to placebo in patients with onchocerciasis, loiasis, mansonellosis and/or the intestinal whip-worm infection trichuriasis. The trial is being conducted in the Democratic Republic of the Congo, the Gabonese Republic, the Republic of Cameroon, and the United Republic of Tanzania and started in June 2025. To support planning for the eWHORM study, we conducted a systematic review of RCTs in onchocerciasis, loiasis, and mansonellosis, focusing on their trial design. Our primary aim was to provide a comprehensive overview of the statistical methods used, with emphasis on qualitative or quantitative primary endpoints. Second, we applied the estimand framework introduced in the International Council for Harmonisation guideline, ICH E9(R1) addendum [29], which provides a systematic approach to defining treatment effects in clinical trials, with explicit consideration of intercurrent events such as treatment discontinuation, use of rescue medication, or death. We extracted information on the estimand attributes population, variable (endpoint), summary measure, as well as the strategies to deal with intercurrent events. Additionally, we described the trials with respect to sample size and number of arms, and summarized key statistical parameters relevant for study design, including advanced design features such as interim analyses and multiplicity adjustment. The search and information extraction followed the PRISMA guidelines [30].

## Materials and methods

### Search strategy

The search was conducted in the literature databases PubMed (https://pubmed.ncbi.nlm.nih.gov/) and the four registry databases ClinicalTrials.gov (https://ClinicalTrials.gov/), WHO International Clinical Trials Registry (ICTR) (https://trialsearch.who.int/), International Standard Randomised Controlled Trial Number (ISRCTN) (https://isrctn.com/) registry, and the Pan African Clinical Trials Registry (PACTR) (https://pactr.samrc.ac.za/). The search was limited to RCTs in humans from January 1, 2000 to December 31, 2024 in English or French. The final query in the databases was carried out on January 23, 2025. The search terms used in the individual databases are specified in S1 Table.

### Eligibility criteria

The review addressed RCTs assessing efficacy and/or safety in onchocerciasis, loiasis, and mansonellosis, identified from articles in peer-reviewed journals or registered in the clinical trial registries between January 1, 2000 and December 31, 2024. For trials published as an article, this time frame refers to the publication date. If a trial had not yet been published and only a record in a registry was identified, the time frame refers to the registration date in the registry. We excluded studies focusing solely on new methods, short reports, RCTs in other diseases, and Phase I RCTs, and trials assessing pharmacokinetics.

### Data extraction

All articles or records identified by the search were initially screened based on the title and the abstract (if available) to determine eligibility for the specific disease. In the next step, the full text or the information from the registry was screened for further exclusion. Duplicates of trials identified in PubMed and in a registry were identified via the registry number. Records identified in WHO ICTR were all either duplicated in ClinicalTrials.gov, ISRCTN, or PACTR and linked via the same registry number. After the identification of duplicates, the final set of included trials was determined. The selected articles and/or records were screened and the relevant information was collected in a spreadsheet. For some trials the study protocol was also available from the registry or provided as supplementary material in the article and additional information from the study protocol was retrieved. If the study protocol and article had different information, the information was retrieved from the article. The first author conducted the initial search, applied the exclusion criteria, and screened the identified articles. One additional reviewer performed an independent assessment. Consistency between independent reviewers was evaluated by consensus review to resolve any discrepancies.

### Variables

To extract information from the trials we applied the estimand framework as introduced in the ICH E9(R1) addendum [29] focusing on the description of the attributes population, variable (endpoint), strategies for handling intercurrent events, and summary measure. Although only a small number of the selected trials explicitly referenced the estimand framework, the articles, registries and/or study protocols were systematically examined to identify and extract these attributes. As described in the estimand framework, population was described by the disease and specific inclusion and exclusion criteria (lower and upper limits for mf, age, and weight). For the intercurrent events, we inferred the estimand strategy based on the analysis population used - intention-to-treat (ITT) or per protocol (PP) - when explicitly stated. If the specification of ITT or PP could not be extracted (by the corresponding words), it was recorded as missing. We did not extract specific intercurrent events such as death, treatment discontinuation or compliance. The summary measure describes how outcomes were aggregated and compared between treatment groups in the primary analysis. This included statistics such as arithmetic or geometric means, medians or risk differences. As this was inconsistently reported across studies, we provide an overview of applied descriptive statistics. The attribute variable refers to the primary endpoint and it was categorized in each trial as a qualitative or quantitative variable based on either follow-up values or changes from baseline to follow-up. We also recorded the duration of follow-up used for the primary endpoint. The treatment attribute includes types of intervention(s) and control(s).

In addition to the estimand-related data, we extracted general trial characteristics such as article title, first author, publication/registration year, study objective, registry number, number of arms, country, planned/calculated and actual randomized sample sizes, randomization method, percentage of missing data for the primary analysis, handling of missing data, latest follow-up, the statistical methods applied for the primary endpoints, implementation of interim analyses, and multiplicity control. The full list can be found in S2 Table.

The planned sample size was determined either from the sample size calculation provided in the publication, the study protocol or the reported enrollment number listed in the registry. The actual sample size refers to the number of participants randomized. Again, information was only extracted if the information was explicitly reported. For randomization methods, the information was recorded as unclear if randomization was mentioned in the title or abstract but the method of randomization was not specified. For trials reporting multiple follow-up time points for the primary endpoint, we recorded the maximum follow-up duration.

In general, information on attributes or other study parameters was only extracted when explicitly specified in the publication, the registry and/or the study protocol. Otherwise the parameter was recorded as missing.

A protocol of the systematic review is available as supporting information S1 Text. The review was not registered. Excel files (i) of all identified articles and/or records in registries with reasons for exclusion are provided in S1 Data and (ii) of all 44 included trials and the extracted information in S2 Data. The Excel files are also available at: https://gitlab.com/mougeni/ewhorm_scientificreview.

### Data analysis

Main characteristics were shown for each trial (registration number, primary objective, phase, type of blinding, number of arms, whether a sample size calculation was reported, actual sample size, analysis population, title of article/registry, primary endpoint, intervention and control details). Study characteristics were summarized using descriptive statistics, mainly absolute and relative frequencies of characteristics were computed for each disease and across diseases and for the time periods 2000-2012 and 2013-2024. For planned sample sizes and number of arms, medians and interquartile ranges (IQR) were reported. Actual sample sizes of participants randomized were depicted by boxplots for control and intervention groups per disease, and further stratified by type of control group (placebo/no treatment versus standard drug/dose). In trials with more than one intervention group, average sample sizes were shown. Additional boxplots summarized follow-up duration (overall and for the primary endpoint) and percentage of missing values by disease. Barplots were used to show the frequency of statistical methods applied to quantitative and qualitative endpoints overall and for time periods 2000-2012 and 2013-2024. R version 4.3.1 (R Foundation for Statistical Computing, Vienna, Austria) was used for all statistical analyses and plots.

## Results

**Fig 1:**
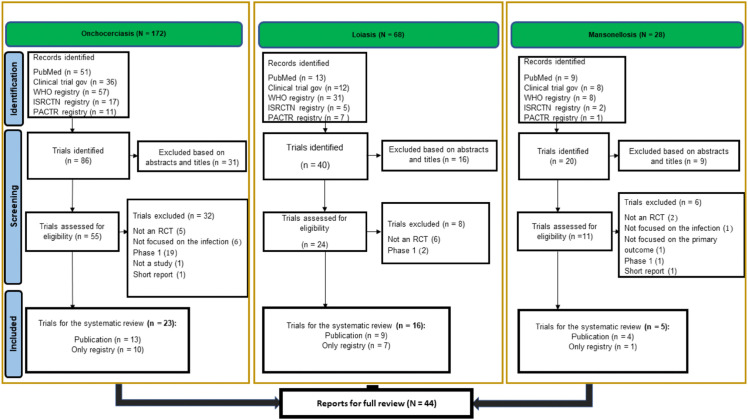
PRISMA flow chart for study selection process per disease.

We identified 268 articles and/or records in registries (Fig 1). Overall, 122 reports were removed as duplicates. From the 146 remaining reports, 56 records were removed based on the abstract or title. Furthermore, 46 reports were removed from the remaining set of 90 studies for not meeting the inclusion criteria (see S1 Data). The remaining 44 trials were included in the systematic review and the full text was assessed (23 (52%) for onchocerciasis, 16 (36%) for loiasis, and 5 (11%) RCTs for mansonellosis). One identified trial was the eWHORM basket trial comprising 3 substudies on onchocerciasis, loiasis, and mansonellosis. These substudies were treated as independent trials and assigned to the corresponding diseases.

### Study designs and analysis populations

**Fig 2:**
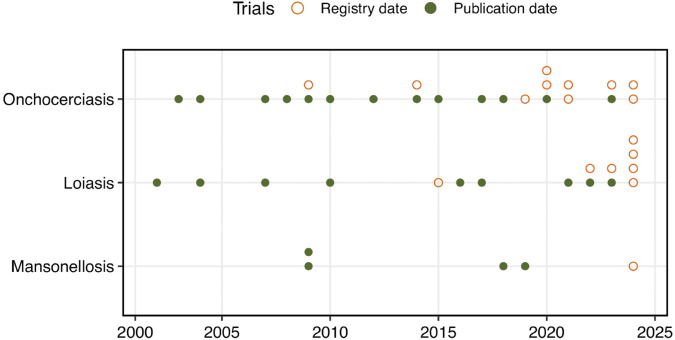
Timing of publication/registry data. The 44 identified trials by year of publication, or by registration year if no publication was available (empty circles).

Table 1 summarizes the key characteristics of the 44 identified trials, S3 Table lists the titles of the articles/registries and the primary endpoints (all extracted information can be found in S2 Data). For some trials, information was incomplete. For 18 (41%) of trials no publication was available, and several parameters were missing such as study phase, whether a sample size calculation was performed (sample size calculation), actual sample sizes of participants randomized, or analysis population. Among these 18 trials, 3 provided a study protocol or a statistical analysis plan (SAP) and one master protocol was available for the 3 eWHORM trials. In 10 trials with a published manuscript, no registration number was found in the full text. Matching these articles with registry entries was limited due to inconsistencies in registry identifiers, as the intervention name or study design alone often led to ambiguity or potential mismatches. Thus, only records with the same registry number were matched. For 16 trials (36%) both the published manuscript and a registry number were available (note that for earlier trials registration was not mandatory). Fig 2 shows the identified trials by publication year (or registration year if unpublished) for each disease.

**Table 1:** The 44 identified trials and their main characteristics.

Nr	Registration number	Primary objective	Phase	Blinding	Nr. of arms	Sample size calculation	Actual sample size	Analysis population	
**Onchocerciasis**									
1	NCT04188301	safety & efficacy	-	open-label	3	yes	154	-	[31]
2	ISRCTN50035143	efficacy	-	open-label	4	yes	294	ITT	[2]
3	NCT00790998	efficacy	3	double-blind	2	yes	1472	ITT	[1]
4	NCT00300768	safety	2	double-blind	4	yes	172	ITT	[32]
5	ISRCTN48118452	efficacy	-	double-blind	3	yes	150	-	[33]
6	-	not clear	-	open-label	2	-	240	-	[34]
7	ISRCTN66649839	efficacy	-	double-blind	2	yes	167	PP	[35]
8	-	efficacy	-	double-blind	3	-	42	-	[36]
9	ISRCTN06010453	efficacy	2	open-label	5	-	123	PP	[37]
10	-	efficacy	-	-	4	yes	657	PP	[38]
11	ISRCTN71141922	efficacy	-	double-blind	3	yes	76	-	[27]
12	-	not clear	-	single-blind	2	-	12	-	[39]
13	-	not clear	-	open-label	4	yes	78	-	[40]
14	ISRCTN68861628	efficacy	-	double-blind	5	yes^*^	400	-	[37]
15	NCT06070116	safety & efficacy	2	open-label	4	-	-	-	[41]
16	NCT04913610	efficacy	2	double-blind	5	yes	-	PP	[42]
17	NCT02078024	efficacy	3	open-label	5	-	300	-	[43]
18	NCT05180461	efficacy	2	double-blind	5,3	-	-	-	[44]
19	ISRCTN43697583	efficacy	2	open-label	4	yes^*^	-	-	[45]
20	ISRCTN38954299	efficacy	2	open-label	7	yes^*^	-	-	[46]
21	NCT03876262	safety & efficacy	3	double-blind	4	yes	-	-	[47]
22	PACTR202009704006025	efficacy	2	open-label	4	-	-	-	[48]
23	PACTR202412611774752	efficacy	2	double-blind	4	yes*	-	ITT	[49]
**Loiasis**									
24	NCT04049630	safety	2	double-blind	4	yes	255	-	[50]
25	ISRCTN25831558	efficacy	-	double-blind	3	yes	60	ITT	[24]
26	PACTR201807197019027	efficacy	-	assessor-blind	4	yes	46	PP	[51]
27	-	not clear	-	single-blind	2	-	95	-	[25]
28	-	not clear	-	open-label	5	-	80	-	[52]
29	-	not clear	-	double-blind	3	-	95	PP	[53]
30	-	not clear	-	double-blind	2	-	99	-	[54]
31	NCT01593722	efficacy	4	open-label	2	-	12	-	[55]
32	NCT01111305	efficacy	2	double-blind	2	yes	8	-	[56]
33	NCT06252961	safety	2,3	double-blind	3	-	-	-	[57]
34	NCT02644525	efficacy	2	double-blind	4	-	20	-	[58]
35	NCT04049851	safety	2	double-blind	2	-	-	-	[59]
36	NCT06613997	safety & efficacy	3	double-blind	2	-	-	ITT	[57]
37	PACTR202303704849277	safety	2	assessor-blind	3	-	-	-	[60]
38	PACTR202412611774752	efficacy	2	double-blind	4	yes*	-	ITT	[49]
39	PACTR202411787280874	safety	2	double-blind	4	-	-	ITT	[61]
**Mansonellosis**									
40	-	efficacy	3	double-blind	2	yes	-	-	[62]
41	NCT02281643	efficacy	2	open-label	2	-	202	PP	[18]
42	NCT00340691	efficacy	2	open-label	2	yes	230	-	[17]
43	NCT00215280	efficacy	-	double-blind	2	-	182	-	[63]
44	PACTR202412611774752	efficacy	2	double-blind	4	yes*	-	ITT	[49]

Sample size calculation refers whether a sample size calculation was reported for the trial; the actual sample size is the number of participants randomized. For trials with numbers 14-23, 33-39, and 44 only a registry was available, for trials 1, 4, 16, 21, 23, 25, 26, 34, 38, and 44 a study protocol or a statistical analysis plan was available. Analysis population was based on intention-to-treat (ITT) or per protocol (PP).

* Information obtained from authors.

**Fig 3:**
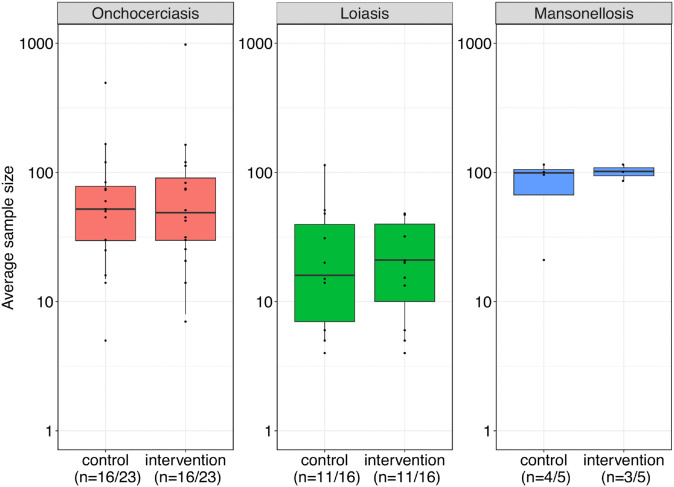
Boxplots and individual data points of actual sample sizes (number of participants randomized) for control and intervention groups for each disease. The lines relating the points in each plot reflect the ratio of the group sample size in a specific trial. A log10 scale was used in the boxplot to enhance visualization. Note: The whiskers extend from the box to the minimum and maximum values excluding outliers. Outliers, defined as 1.5 times the interquartile range away from the box, are represented by points only.

All trials tested a superiority hypothesis. A planned sample size was reported in 36 trials (82%) derived either from the sample size calculation presented in the article or the target sample size reported in the registry. In these trials, the median total number of planned participants was 166 (IQR: 82-240). However, only 23 trials (52%) including 15 with a published manuscript reported a formal sample size calculation (for 6 trials this information was obtained from the authors). For loiasis, smaller total planned sample sizes were observed (median 99, IQR: 60-160) compared to the other diseases (mansonellosis: median 180, IQR: 105-200, onchocerciasis: median 220, IQR: 153-323). Fig 3 shows the actual sample sizes per arm for the control and intervention groups for the primary endpoint, stratified by disease. In trials with multiple intervention groups, average sample sizes are shown. Most trials used a 1:1 allocation ratio.

Information on trial phase was available for 26 trials. Reported phases were Phase 2 (19 trials), Phase 2/3 (1 trial), Phase 3 (5 trials), and Phase 4 (1 trial). For some trials, the registries listed the phase as “not applicable”. Regarding the analysis population for the primary analysis, 7 trials used PP and 9 used ITT analysis. In total, 64% of the trials did not specify the type of analysis population (see Table 1).

Twenty-two trials (50%) reported inclusion criteria based on mf counts. Among these, 9 trials specified both lower and upper mf thresholds. For loiasis, 75% of trials reported mf-based inclusion criteria, whereas the corresponding proportions were lower for onchocerciasis (39%) and mansonellosis (20%). Detailed lower and upper bounds for mf for each trial as well as bounds for age and weight are shown in S4 Table.

#### Interventions.

In the 44 trials, different interventions were evaluated, including IVM, ALB, levamisole, DEC, DEC-medicated salt, moxidectin, a combination of quinine, chloroquine, amodiaquine, and artesunate, emodepside, reslizumab, rifampin, azithromycin, DOXY and others (for details on each study, see S5 Table). The number of study arms, including the control group, varied by disease: loiasis and mansonellosis trials typically had two or three arms (median 2 for mansonellosis and 3 for loiasis), while onchocerciasis trials often had more than three arms (median: 4, IQR: 3-4). Placebo was the most frequent control in loiasis (10/16) but not in onchocerciasis studies, where a standard drug/dose was most frequently reported as control (15/23 trials). For mansonellosis, placebo and standard drug/dose were each reported twice (see Table 2). Control groups receiving “no treatment” were reported in two trials in the earlier time period (2000-2012), but were not observed in the later period (2013-2024) (see S6 Table).

**Table 2:** Frequency table with details of control group and method of randomization overall and for each disease.

Characteristic	Overall N = 44	Onchocerciasis N = 23	Loiasis N = 16	Mansonellosis N = 5
**Control group**				
No treatment	2 (4.5%)	0 (0%)	1 (6.3%)	1 (20%)
Placebo	20 (45%)	8 (35%)	10 (62%)	2 (40%)
Standard drug/dose	22 (50%)	15 (65%)	5 (31%)	2 (40%)
**Randomization**				
Simple randomization	3 (6.8%)	2 (8.7%)	1 (6.3%)	0 (0%)
Block randomization	7 (16%)	1 (4.3%)	5 (31%)	1 (20%)
Stratified randomization	14 (32%)	9 (39%)	5 (31%)	0 (0%)
Not specified	20 (45%)	11 (48%)	5 (31%)	4 (80%)

#### Randomization and blinding.

Among the 44 trials, 24 (55%) reported details on the randomization procedure (Table 2). Stratified randomization was the most frequently reported method (14 trials, 32%), followed by block randomization (7 trials). Note that all trials with block randomization were from time period 2013-2024 (S6 Table). Stratification was commonly based on mf load, other stratification factors included age, sex, and mf load, or status of mf[1,27,35].

Double-blind designs predominated (25/44, 57%) (see Table 1). Open-label trials accounted for nearly one-third of studies (14/44, 32%), whereas single-blind and assessor-blind designs were rare (each 4/44, 9%). Blinding was unspecified in one trial (2.3%). By disease, double-blinding was most frequent in loiasis trials (11/16, 69%), followed by mansonellosis (3/5, 60%) and onchocerciasis (11/23, 48%). Open-label designs were particularly common in onchocerciasis (10/23, 43%) and mansonellosis (2/5, 40%), but less frequent in loiasis (2/16, 13%).

### Primary objectives and endpoints

The primary objective was defined for 37 trials (see Table 1), either in the methods sections of the manuscripts or the registries. Of these, 33 trials had a single primary objective, which was related to efficacy in 27 and to safety in 6 trials. In further 4 trials, both efficacy and safety were defined as the primary objective. In the remaining 7 trials, the definition of the primary endpoint was missing. Further details on the specification of the primary endpoint can be found in S3 Table.

Among the 31 trials with a primary efficacy endpoint, 14 trials defined a quantitative endpoint, 15 a qualitative endpoint, one trial reported both (estimand attribute variable/endpoint), and one was not clearly defined (Table 3). Qualitative endpoints were reported more frequently than quantitative endpoints for onchocerciasis trials only.

**Table 3:** Types of efficacy endpoints, primary efficacy endpoints and primary efficacy endpoints focusing on mf

Type of endpoint	Overall N = 44	Onchocerciasis N = 23	Loiasis N = 16	Mansonellosis N = 5
**Efficacy endpoint**				
Qualitative	17 (39%)	12 (52%)	4 (25%)	1 (20%)
Quantitative	21 (48%)	8 (35%)	9 (56%)	4 (80%)
Both	2 (4.5%)	0 (0%)	2 (13%)	0 (0%)
Not available/not clear	4 (9.1%)	3 (13%)	1 (6%)	0 (0%)
**Primary efficacy** endpoint				
Qualitative	15 (48%)	12 (63%)	2 (29%)	1 (20%)
Quantitative	14 (45%)	6 (32%)	4 (57%)	4 (80%)
Both	1 (2.3%)	0 (0%)	1 (14%)	0 (0%)
Not available/not clear	1 (2.3%)	1 (2%)	0 (0%)	0 (0%)
**Primary efficacy endpoint based on** mf				
Qualitative	6 (14%)	3 (13%)	2 (13%)	1 (20%)
Quantitative	10 (23%)	4 (17%)	2 (13%)	4 (80%)
Both	1 (2%)	0 (0%)	1 (8%)	0 (0%)

In 17 trials, the primary efficacy endpoint was defined based on measurement of mf values. Of these, 6 trials assessed mf qualitatively, for example by evaluating whether participants reached a pre-specified threshold or fell below a defined mf level. Ten trials assessed mf quantitatively as primary endpoint and one trial used both. Among the trials with a quantitative primary endpoint, five trials focused on the changes in mf from baseline to follow-up, whereas 6 assessed mf values at follow-up (results comparing the time periods 2000-2012 and 2013-2024 can be found in S7 Table).

**Fig 4:**
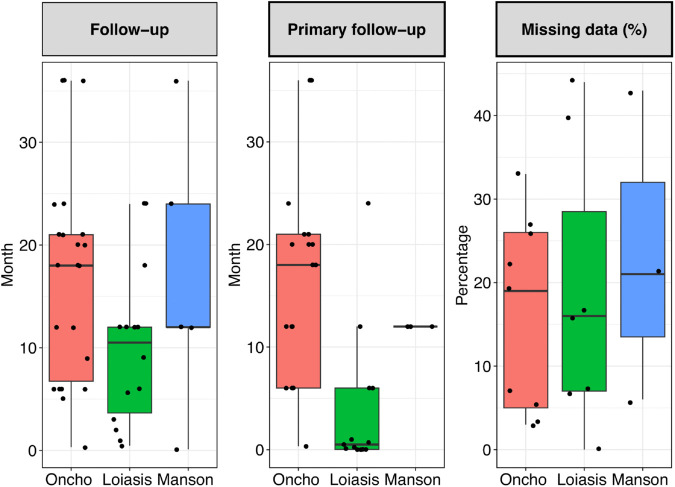
Boxplots showing complete follow-up, follow-up for the primary endpoint and percentage of missing data. Numbers are shown for onchocerciasis (Oncho), loiasis and mansonellosis (Manson). Percentage missing data were either directly reported for some trials or calculated as the difference between the number of patients initially randomized and those who remained at the end of the follow-up period for the primary endpoint.

Fig 4 shows boxplots of follow-up times for the primary endpoints and the complete follow-up of the trials, along with the percentage of missing data at the primary endpoint time point. The follow-up durations (both for complete and primary endpoint) were generally shortest for trials investigating loiasis.

### Statistical methods

#### Statistical methods for quantitative endpoints.

**Fig 5:**
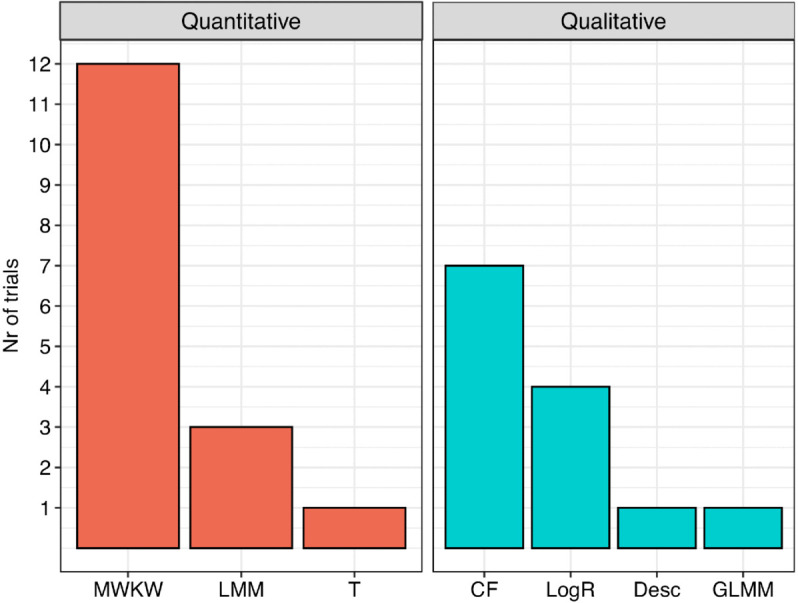
Frequency of statistical methods applied to quantitative and qualitative efficacy endpoints. The quantitative methods are the non-parametric Mann-Whitney U or Kruskal-Wallis test (MWKW), the parametric t-test (T), linear mixed model or repeated measure analysis of variance (LMM). The qualitative methods are descriptive only (Desc), chi-squared or Fisher’s exact test (CF), logistic regression (LogR), and generalized linear mixed model (GLMM).

The most commonly reported inferential methods for quantitative efficacy or safety primary endpoints were univariable analyses (≥70%), whereas multivariable methods were only used in approximately 25% of the trials. For quantitative primary endpoints, the Mann-Whitney U or Kruskal-Wallis tests (MWKW) were the most frequently applied methods (12 trials), followed by linear mixed models (LMM) (see Fig 5 and S1 Fig, stratified by time period). Two other trials used a generalized linear mixed model (GLMM) for quantitative endpoints of either a secondary (efficacy) endpoint or an endpoint which was neither primary or secondary. In these models, random effects included study participant (to adjust for multiple worms per person) or study site. Fixed effects included baseline mf, level of infection, sex, treatment, and treatment by covariate interactions.

Results of the analyses for the quantitative primary endpoints were reported by several summary measures within studies, such as geometric and arithmetic mean and median. However, summary measures and analysis methods were often not aligned. A primary summary measure was only defined in 11 trials (geometric mean in 6, median in 2, and concordance in 3 trials). The concordance (also called probabilistic index or relative effect [64]) is a summary measure for the Mann-Whitney U test and was defined as summary measure in the eWHORM substudies [49].

#### Statistical methods for qualitative endpoints.

For trials with primary qualitative endpoints, the chi-squared or Fisher’s exact tests were the most commonly used methods (7 trials), 4 trials reported logistic regression and 1 generalized linear mixed model (see Fig 5). The summary measure proportion for the analyses was explicitly defined in 4 trials.

#### Multiplicity adjustment.

Although 30 trials had more than two arms, only six reported methods to control the familywise error rate (FWER): Two trials applied the Bonferroni correction [24,53]. One trial used a closed testing procedure to control the FWER which involved a global test of equal rates of fertile female worms for four treatments and all intersection hypotheses considering three treatments at level *α*, followed by pairwise intersection hypotheses tested at level α/2 [2]. In the three eWHORM substudies a partial conditional error approach was embedded within a closed testing procedure to ensure strong control of the FWER for the multiple doses and the two stages [49]. One trial planned to conduct an interim analysis after all patients had been recruited and had reached their 3 month follow-up. Based on these interim data, the total length of follow-up was planned to be re-evaluated [58].

#### Handling of missing data.

Several trials reported approaches to handle missing data for the primary endpoint. One trial applied the “extreme analysis” to assess the significant effect of the intervention evaluating the robustness of the treatment effect under a worst-case assumption [17]. Other approaches were linear interpolation to impute the missing values [25], a log-rank test to compare the drop-out patterns between the groups over time [33], assumption of a worst-case scenario [35], and multiple imputation using logistic regression methods [42]. In [47] sensitivity analyses for the primary efficacy endpoint were proposed to explore the impact of various imputation algorithms on the primary efficacy endpoint. In the eWHORM substudies missing data were planned to be handled by multiple imputation methods specifically tailored for zero-inflated data [65].

### Clinical trial examples

Below we summarize the latest publication for each disease to give an overview of the trials conducted and we describe the eWHORM trial.

#### Onchocerciasis: IVM, DEC, and ALB [31]:

The primary safety objective of this randomized, parallel-group, open-label trial was to compare frequencies and types of severe adverse events (grade 3 or higher) occurring within 7 days following either a 1 day or a 3 days regimen with the triple drug IDA (DEC in combination with IVM and ALB). The control group received a 1 day treatment of IVM and ALB only. All groups were pretreated with IVM to clear mf and to ensure the safety of the DEC combination treatment. The study population consisted of individuals aged 16 to 70 years with onchocerciasis, and a baseline skin mf count of > 3 mf/mg. Stratified randomization was performed based on the presence of ocular mf. A total of 154 participants were randomized. The phase of the trial was not specified. The primary estimand for efficacy was defined as the difference between treatment groups in the proportion of adult female *O. volvulus* worms that remained alive with embryos in the uterus 18 months after treatment. This endpoint targeted the effect of each regimen on worm sterilization or death, assessed through histological examinations of adult female worms extracted from nodules of treated individuals. Odds ratios for the comparison with the control group and 95% confidence intervals were reported using a mixed-effects logistic regression model to account for clustering of worms within individual participants. Secondary estimands for efficacy included the comparison of log-transformed mf counts between groups at 18 months, analyzed using analysis of variance (quantitative endpoint) and comparison of mf presence (yes/no) between groups analyzed by Fisher’s exact test (qualitative endpoint). For primary safety endpoints, frequencies of adverse events were compared across the three treatment groups using chi-squared and Fisher’s exact test and proportions were reported. The findings indicated that the IDA regimens were well tolerated and more effective compared to the control group.

#### Loiasis - ALB and IVM [51]:

This randomized, assessor-blinded, controlled clinical trial evaluated the efficacy and safety of three ALB-based treatment regimens compared to the control group receiving symptomatic treatment with the antihistamine drug loratadine. All treatment groups started with three weeks of ALB followed by either no treatment, two further weeks of ALB, or a single dose of IVM. The study aimed to assess the efficacy of ALB and IVM to reduce *L. loa* mf. The target population of interest were adult participants in Gabon with baseline mf between 5000 and 50000 mf/ml. Block randomization was performed for unequal allocation across groups with a 1:2:2:2 ratio for control versus the three treatment arms. A total of 46 participants were randomized. The phase of the trial was not specified. The primary estimand focused on the proportion of patients achieving a mf load below 100 mf/ml within 6 months under the assigned treatment regimen. The PP population was used for the primary efficacy analysis including one participant who inadvertently switched treatment group. No rescue medication was reported. The primary analysis was descriptive with proportions and odds ratio with confidence intervals. ALB administered for 5 or for three weeks followed by a single dose of IVM appeared to be the most effective treatment regimens achieving the primary endpoint in 39% and 22% of participants, respectively. No participant from the control group reached the primary endpoint. IVM was administered only after assurance that *L. loa* mf load was below 4000 mf/ml to minimize potential risks associated with the use of IVM in microfilaremic individuals.

#### Mansonellosis - DOXY [18]:

This open-label, randomized Phase 2 trial investigated (i) the presence of *Wolbachia* bacteria in *M. perstans* in Ghana and (ii) whether DOXY treatment would deplete *Wolbachia* and cause a slow, sustained decline in mf. The study enrolled participants aged 10 to 55 years, inclusion criteria based on mf status were not clearly specified. The participants were 1:1 randomized into two groups. The first, immediate group received treatment after randomization while the delayed arm received the same treatment 6 months later. A total of 202 participants were randomized. The primary estimand targeted the difference in *M. perstans* mf load at month 12 between the two treatment groups, analyzed using both the ITT and the PP populations. The Mann–Whitney U-test was performed to compare groups and geometric mean and median were reported as summary measures. The findings showed that a 6 week course of DOXY effectively induced long-term reductions of mf in a Ghanaian population infected with *M. perstans*.

#### Onchocerciasis, loiasis and mansonellosis - oxfendazole (eWHORM):

The eWHORM study is an adaptive, Phase 2, randomized controlled clinical basket trial to evaluate the efficacy and safety of oxfendazole compared to placebo in patients with trichuriasis, onchocerciasis, loiasis and/or mansonellosis. The basket trial design included a shared master protocol with separate efficacy evaluations for each disease arm. For onchocerciasis, loiasis and mansonellosis an interim analysis was planned to be conducted for dose selection and potential sample size reassessment. The basket arm for trichuriasis has already been completed, the three other basket arms started in 2025 or will start in 2026. While efficacy will be assessed independently for each basket, (interim) data from all diseases may be utilized for safety assessments and dose selection during interim analyses. Several estimands for the primary and secondary objectives were defined in the master protocol and the SAP. The primary objective was to identify a safe and efficacious dose of oxfendazole for each of the four indications, the primary endpoint was parasite load (mf load for onchocerciasis, loiasis and mansonellosis) at month 12 or day 18 (for trichuriasis). The primary analysis of the primary objective was planned to be based on a modified ITT (mITT) population, defined as the population including all participants who received at least one intake of the investigational product and for which the primary endpoint at Month 12 was observed or who died or took rescue medication (treatment policy strategy). The primary estimand for each considered dose compared to placebo was described by the attributes defined in Table 4. The secondary estimand of the primary objective was planned to be based on a PP population defined as the population including all participants in the mITT population who were free from major protocol violations that could lead to bias of results (hypothetical strategy). The summary measure for the primary estimand was the concordance of parasite load between intervention conditions and placebo, the analysis was planned to be performed with one-sided Mann–Whitney U tests controlling the FWER at the 2.5% level. The trial incorporated a partial conditional error approach to accommodate the potential inclusion of a new arm after the interim analysis [66].

**Table 4:** Primary estimand of primary objective of eWHORM study as described in the master protocol

Attributes	
**Treatment**	Oxfendazole and placebo
**Population**	Participants with onchocerciasis/loiasis/mansonellosis as described by the inclusion and exclusion criteria with at least 1 dose of study drug and Month 12 result for the endpoint (mITT population).
**Endpoint**	Parasite load (mf count) at Month 12
**Intercurrent Events (ICE)**	
ICE 1	Treatment discontinuation, non-compliance.
	a) Regardless of treatment discontinuation (e.g., discontinuation by the investigator if he/she judges that treatment is no longer appropriate, if the participant’s clinical condition is worsening, or for AE) a treatment policy strategy will be used to estimate treatment effect. I.e., also for patients with treatment discontinuation, the estimand takes the value of the primary endpoint (parasite load at Month 12).
	b) Regardless of non-compliance to the study treatment and/or background medication or discontinuation or change in dose regimen of study treatment and/or background medication, a treatment policy strategy will be used to estimate treatment effect. I.e., also for patients with non-compliance, the estimand takes the value of the primary endpoint (parasite load at Month 12).
ICE 2	Death, rescue medication.
	A hypothetical strategy will be used to estimate what the treatment effect would have been if participants did not die or receive rescue medication. That is, a worst outcome scenario will be assumed, where the highest possible rank in the primary outcome variable is assigned to participants who died or received rescue medication.
**Population-level summary**	Concordance of parasite load between placebo and intervention conditions at Month 12

## Discussion

This systematic review provides an overview of the trial designs and statistical methodologies used in RCTs investigating treatments for onchocerciasis, loiasis, and mansonellosis from 2000 to 2024. The findings revealed considerable heterogeneity across trials: they differed not only in study-specific parameters such as the number of arms or control groups, but also in trial design parameters that could potentially be harmonized within each disease or even across diseases for trials with similar objectives. These include the definition of the primary endpoint (including the length of the follow-up period and endpoint type, qualitative or quantitative), the corresponding analysis method, and the choice of analysis population within each disease.

Some of the observed heterogeneity may be due to the mode of action of the different drugs, the filarial species investigated, and the life cycle stages being targeted. However, a harmonisation of endpoints would facilitate the comparison of data across trials as well as joint analyses. Initiatives such as eWHORM, which addresses multiple diseases within a single master protocol aim to standardise the conduct and analysis of clinical trials for helminth infections.

Onchocerciasis, loiasis, and mansonellosis are rooted in complex biological processes, involving tissue-dwelling nematodes that develop within a vector, resulting in multiple changes in host, location, and habitat throughout their life cycle. Furthermore, for mansonellosis and loiasis, capturing and measuring the adult worm burden (which is critical for diagnosis and determining cure rates) poses significant challenges. The interdependencies among worms, larvae, and newly acquired worms, as well as their distribution cycles, remain largely unexplored. Nevertheless, there is an urgent need for improved treatments [13], and these challenges should not hinder efforts to evaluate new drugs and drug combinations, using appropriate statistical methods to identify new effective treatment options.

This review showed that the majority of trials were double-blind to minimize bias, in line with established guidelines [67]. Stratified randomization was the most common approach, followed by block randomization. Stratification is especially valuable in trials with small sample sizes when prognostic factors are known [68]. This is often the case in parasitic diseases, where baseline parasite burden can impact the treatment response, for example in areas with a history of repeated MDA compared with MDA naive areas [2,31,53,69,70]. Simple randomization, where participants are assigned to treatment arms with equal probability regardless of their baseline characteristics, can prevent selection bias [71]. However, in small trials it may result in poor covariate balance and potentially biased treatment effect estimates [72]. This is particularly relevant in filarial disease research, where trials with larger sample sizes are uncommon because of budgetary constraints. Consequently, alternatives such as stratified permuted block randomization with a limited number of stratification factors are often recommended to reduce imbalance in small trials and analyses that incorporate the stratification variables are advocated [73–76].

According to the International Council for Harmonisation guideline ICH E10 [77], a control group is used to distinguish outcomes attributable to the study treatment from changes due to the natural course of the disease over time, or other non-treatment factors such as expectations and concomitant care. Without a control group, treatment effects may be overestimated, in particular if eligibility is based on high baseline mf counts because of regression to the mean. Regression to the mean describes the tendency for participants selected based on high mf counts to have values closer to the population mean at follow-up, even without effective intervention [78]. Therefore, inclusion and exclusion criteria based on mf counts can substantially influence quantitative outcomes and their interpretation. Overall, 50% of the trials reported mf-based inclusion criteria, most frequently in loiasis trials (75%). This is consistent with safety concerns, because patients with high mf loads are at increased risk of severe adverse events when treated with microfilaricidal drugs such as IVM or DEC. This necessitates excluding participants with elevated mf counts. Accordingly, 9 out of the 16 loiasis trials had an upper mf threshold whereas only three used a minimum mf count alone. In onchocerciasis and mansonellosis trials upper mf limits were uncommon, and a minimum mf count was applied in 39% and 20% of trials, respectively. While mf-based inclusion criteria are often justified by safety considerations, they may increase regression-to-the-mean effects. For example, if eligibility requires mf larger than a threshold at baseline, many participants will show lower values at follow-up due to natural variability, even without effective treatment. Without a control arm, the resulting regression-to-the-mean effect cannot be separated from true treatment effects.

The choice between placebo or no treatment controls and active or dose-response controls depends on the trial objectives and ethical considerations. Placebo controlled designs are often more efficient in early-phase trials aimed at establishing proof of concept, because larger relative effect sizes can reduce required sample sizes. In contrast, in MDA and elimination programs, active controls may improve external validity and provide evidence that is more directly relevant for programmatic decisions and policy implementation. Placebo controls are not ethically justifiable when withholding an established, safe and effective treatment could result in death or irreversible morbidity [77]. In this review we identified both active-controlled and placebo or no treatment controlled trials across all three diseases. As expected, trials with a standard drug or dose in the control group generally had larger sample sizes (median 51) than trials with placebo or no treatment controls (median 26, see boxplots comparing trials where control is a placebo versus trials where control is a standard drug/dose for each disease in S2 Fig).

For most trials, the primary objective was to show efficacy and both, qualitative and quantitative primary endpoints were chosen. Quantitative endpoints more efficiently make use of the available information in the data than derived dichotomized variables. For example, mf counts are more sensitive measures than the derived binary response variable “-50% decrease in mf”. In onchocerciasis trials, the primary endpoints were mainly qualitative. This might reflect the specific clinical goals of these studies, where achieving a clear-cut therapeutic outcome (e.g., absence of mf) is important [37,79]. To investigate the macrofilaricidal activity, drug effects on the fertility and viability of adult worms are most relevant. Unfortunately, the direct analysis of adult worms is often not feasible or lacks evaluation of pretreatment status, particularly for loiasis and mansonellosis, where adult worms are in the subcutaneous tissues and serous cavities, respectively, and are difficult to locate and retrieve. As a result, many studies rely on mf counts as an indirect measure of treatment effect.

Mf counts often follow a so called mixture distribution, with a positive probability of zero values and a positive component resembling a count distribution such as a Poisson or Negative Binomial distribution (here, “mixture” refers to the outcome distribution at a given time point and is unrelated to the mixed-effects models). Clinically even an mf count of zero does not imply the complete elimination of adult worms. Adult worms may persist and later resume reproduction. Therefore, a reduction of mf loads does not guarantee that the adult worms have died or are permanently sterile. Despite this limitation, mf counts remain relevant because symptoms and transmission in onchocerciasis are mostly caused by mf, not the adult worms and, as mentioned above, the adult worms for loiasis and mansonellosis cannot be assessed directly. To model such a mixture distribution of mf counts, several modeling strategies are available. Two-part models separately analyse the probability of zero versus non-zero values and the distribution of positive counts [80,81]. Other options include zero-inflated Poisson models [82,83], or zero-inflated negative binomial models. None of the 44 trials performed or planned such approaches. Instead, most trials with quantitative endpoints relied on univariable rank-based non-parametric methods, which do not rely on strong distributional assumptions. While these methods are often robust to zero-inflation and control the type I error rate also for small sample sizes, they use only the ordering of observations rather than the observed mf counts, which can reduce efficiency and limit clinical interpretability. In addition, such univariable analysis methods can reduce statistical power and result in more variable treatment effect estimates when prognostic covariates are available. Multivariable methods, such as multiple linear or logistic regression, allow simultaneous adjustment for multiple covariates [84].

Longitudinal data are common in clinical trials for parasitic diseases, where measurements are collected at multiple time points. Mixed-effects models can jointly analyse such data by accounting for within-subject correlation. Despite these advantages, mixed-effects models were reported in only three trials. For longitudinal zero-inflated endpoints, two-part mixed-effects models (also called zero-inflated mixed models) have been proposed [85–88]. Extending two-part models for a single time point, these approaches split the outcome into a binary component for the probability of a zero versus a non-zero value, and a continuous (or count) part for the distribution of the positive observations. Both components can include random effects to account for within-subject correlation from repeated measurements. These models account for the excess zeros while modelling subject-specific trajectories over time. In clinical trials with longitudinal zero-inflated endpoints such as mf counts, these methods may improve estimation, increase power, and support a more clinically interpretable treatment effect compared to non-parametric approaches. However, they rely on parametric assumptions for both the zero component and the distribution of the positive values.

Complex clinical trial designs such as adaptive group sequential designs have not been widely applied in filarial disease trials. Regulatory guidance emphasizes that any adaptations should be accompanied by analysis methods that account for the adaptive decision-making to maintain control of statistical error rates and ensure valid hypothesis testing [89–94]. Adaptive designs can improve efficiency because they allow the trial to be adapted based on interim data, enabling an efficient use of accumulating data. eWHORM is a two stage trial evaluating oxfendazole: it first evaluates two lower doses to establish safety and at interim either adds a higher-dose arm if lower doses are insufficiently efficacious and no safety concerns arise, or continues one or both lower doses. In addition, the eWHORM trial uses a basket design structure, which allows to monitor safety across disease indications and evaluate co-infections.

In several trials identified in this review, multiple interventions were compared to control, but only six reported methods to control the FWER. Several of these trials were described as pilot trials with no intent to perform confirmatory hypothesis testing. However, in confirmatory trials, failing to adjust for multiplicity inflates the probability of false positive findings and can lead to incorrect conclusions about treatment efficacy. Depending on the study design and objectives (dose-finding, subgroup analyses, or multiple primary endpoints), methods such as the Bonferroni adjustment or Dunnett’s test can be applied to control the FWER. To increase power, step-wise multiple testing procedures based on the closed testing principle can be used [95]. Graph-based multiple testing procedures have been proposed to align the multiple testing strategy with trial objectives [96]. Clear justification and transparent reporting of the chosen approach are essential for the interpretation of trial results.

This review identified a preference for reporting geometric means for count outcomes, which can be useful for right-skewed positive data. However, interpreting geometric means is most straightforward when the data follow a log-scale model and treatment effects are naturally expressed on a multiplicative scale. When zeros occur, the handling of zeros needs to be reported because it affects the geometric mean. Furthermore, the geometric mean may be inappropriate for scientific questions that primarily focus on absolute rather than relative changes as in [97]. Then estimands based on the original scale as means or medians are more appropriate. An alternative effect measure is the Mann–Whitney (concordance, or probability of superiority) parameter, interpretable as the probability that a randomly selected treated patient has a better outcome than a randomly selected control patient, counting ties as half wins.

This review was limited by sparse or incomplete reporting for several trials indicating a need for more comprehensive and transparent documentation. A clearer definition of study objectives and analytical strategies could be achieved by adopting the estimand framework outlined in the ICH E9 (R1) addendum on estimands and sensitivity analysis in clinical trials [29] in addition to compliance with the CONSORT statement for reporting RCTs [29,98]. ICH E9(R1) provides a framework to align the design and analysis of a clinical trial to the trial objective, whereas the CONSORT statement provides guidance for the transparent and standardized reporting of RCTs. Among the reviewed trials, only the eWHORM trial explicitly applied the estimand framework. For the remaining trials, we retrospectively retrieved information on the five estimand attributes population, treatment, variable (endpoint), intercurrent events and summary measure, wherever possible. The absence of reported information for a given attribute does not necessarily imply that it was not defined in the trial documents, but rather that it was not included in the sources we reviewed.

A formal risk-of-bias assessment of the included RCTs (for example, using the Cochrane Risk of Bias tool [99,100]) was not performed because our primary objective was to characterize the estimands and the design or analysis choices used in these trials, rather than to evaluate the validity of their treatment-effect estimates. In addition, our review was restricted to publicly available information (articles and trial registries). Key information required for a comprehensive risk-of-bias assessment, such as details on blinding, randomisation, deviations from intended interventions, and handling of missing outcomes, would require information that is often only available in protocols, statistical analysis plans, or clinical study reports. Therefore, our assessment is limited to describing the completeness of reporting in the available sources.

## Conclusions

This systematic review identified both strengths and weaknesses in the design, analysis and reporting of RCTs for onchocerciasis, loiasis, and mansonellosis. Several trials were well planned and comprehensive information was available either in published manuscripts or trial registries. However, for several trials, the reported information was sparse, inconsistent, or incomplete. This underscores the need for standardized, structured, and transparent reporting. Adopting established frameworks such as CONSORT and ICH E9 (R1) estimand approach would improve transparency and strengthen alignment between trial objectives, analyses, and reported conclusions.

A major challenge in trials on onchocerciasis, loiasis, and mansonellosis is the typically small sample size, which increases the risk of baseline imbalance between treatment groups. The use of block and stratified randomization when key prognostic factors are identified can improve balance on important covariates. Proper blinding can reduce bias by helping to avoid systematic differences in care or outcome assessment between arms. In addition, future trials should consider statistical methods aligned to zero-inflated or skewed endpoints, adjustment for covariates, clear definition of the primary summary measure, appropriate handling of missing data, and adjustment for multiplicity when multiple hypotheses are tested.

Finally, adaptive designs can be considered as an option during planning, allowing prespecified design modifications, for example early stopping, sample size re-estimation, or dropping or adding of treatment arms. Whether an adaptive design should be chosen depends on trial objectives as well as practical constraints and should be evaluated on a case-by-case basis.

## Supporting information

S1 Table Database queries. Queries used in PubMed and registries ClinicalTrials.gov, ICTR, ISRCTN, PACTR.
(PDF)

S2 Table Extracted variables in the systematic review. (PDF)

S3 Table Titles and primary endpoints for the 44 trials. (PDF)

S4 Table Selected eligibility criteria. Information on lower and upper limits of mf counts (and corresponding mf units), lower and upper limits of age and weight (sex-specific, if specified) for the 44 trials.
(PDF)

S5 Table Primary endpoint, intervention and control details. Abbreviations: Ivermectin (IVM), diethylcarbamazine (DEC), albendazole (ALB), moxidectin (MOX), doxycline (DOXY).
(PDF)

S6 Table Details of control group and method of randomization overall and for each disease. Frequencies are shown for two time periods 2000-2012 and 2013-2024.
(PDF)

S7 Table Types of efficacy endpoints, primary efficacy endpoints and primary efficacy endpoints focusing on mf. Frequencies are shown for two time periods 2000-2012 and 2013-2024.
(PDF)

S1 Data Data table of identified articles and records. Excel file (.xlsx) shows all identified articles and/or records in registries with reasons for exclusion, also available on gitlab: https://gitlab.com/mougeni/ewhorm_scientificreview.
(XLSX)

S2 Data Data table of extracted variables of included trials. Extracted information of 44 trials provided in xlsx format, also available on gitlab: https://gitlab.com/mougeni/ewhorm_scientificreview.
(XLSX)

S1 Fig Barplot showing frequency of the statistical methods for time periods 2000-2012 and 2013-2024. The quantitative methods are the non-parametric Mann-Whitney U or Kruskal-Wallis test (MWKW), the parametric t-test (TA), linear mixed model or repeated measures analysis of variance (LMM) and the Poisson regression (PR). The qualitative methods are descriptively only (Desc), chi-squared or Fisher’s exact test (CF), logistic regression (LogR), and generalized linear mixed model (GLMM).
(PDF)

S2 Fig Boxplots and individual data points of actual sample sizes comparing trials where the control is placebo or no treatment and trials with control group standard drug/dose for each disease. Note: The whiskers extend from the box to the minimum and maximum values excluding outliers. Outliers, defined as 1.5 times the interquartile range away from the box, are represented by points only.
(PDF)

S3 Fig Boxplots showing complete follow-up, follow-up for the primary endpoint and percentage of missing data. Values are shown for two time periods 2000-2012 and 2013-2024.
(PDF)

S4 Fig Boxplots and individual data points of actual sample sizes (number of participants randomized) for control and intervention groups for each disease. Log-transformed values are shown for two time periods 2000-2012 and 2013-2024.
(PDF)

S1 Text Protocol of systematic review. (PDF)

S2 Text Prisma checklist with manuscript page references. The PRISMA checklist template was obtained from the PRISMA website (https://www.prisma-statement.org/).
(PDF)
