# Supplementary material for: Design and analysis of randomized clinical trials for onchocerciasis, loiasis and mansonellosis: A systematic review
Source: PLoS Negl Trop Dis. 2026 Feb 20;20(2):e0013992. doi: 10.1371/journal.pntd.0013992 (PMC12952602; doi:10.1371/journal.pntd.0013992)
Supplement: S1 Table — Queries used in PubMed and registries ClinicalTrials.gov, ICTR, ISRCTN, PACTR. (PDF) [file pntd.0013992.s001.pdf]

**Type of disease**	PubMed	Other registries
Loiasis	(”loiasis”[MeSH Terms]<br>OR<br>”loiasis”[All Fields]<br>OR<br>”Loa loa”[All Fields])<br>AND<br>((randomizedcontrolledtrial[Filter])<br>AND<br>(2000:2024[pdat]))	loiasis<br>OR<br>Loa Loa<br>OR Loiases
Mansonella	(”mansonella”[MeSH Terms]<br>OR<br>”mansonella”[All Fields]<br>OR<br>”mansonelliasis”[MeSH Terms]<br>OR<br>”mansonelliasis”[All Fields]<br>OR<br>”mansonelliases”[All Fields])<br>AND<br>((randomizedcontrolledtrial[Filter])<br>AND<br>(2000:2024[pdat]))	mansonella<br>OR<br>mansonelliasis<br>OR<br>mansonelliases
Onchocerciasis	”onchocerciasis”[MeSH Terms]<br>OR<br>”onchocerciasis”[All Fields]<br>OR<br>”onchocerciases”[All Fields]<br>OR<br>”onchocerciasis”[MeSH Terms]<br>OR<br>”onchocerciasis”[All Fields])<br>AND<br>((randomizedcontrolledtrial[Filter])<br>AND<br>(2000:2024[pdat]))	onchocerciasis<br>OR<br>onchocerciases<br>OR<br>onchocerciasis
