# Supplementary material for: Design and analysis of randomized clinical trials for onchocerciasis, loiasis and mansonellosis: A systematic review
Source: PLoS Negl Trop Dis. 2026 Feb 20;20(2):e0013992. doi: 10.1371/journal.pntd.0013992 (PMC12952602; doi:10.1371/journal.pntd.0013992)
Supplement: S2 Table — (PDF) [file pntd.0013992.s002.pdf]

Variables collected	Value	Description
Title of the article	[Text]	
Doi	[Text]	link to the publication
Year of publication (registration)	[Integer]	Year of trial publication or registration of the protocol
Registry	[Text]	The registry where the trial was recorded
Inclusion confirmation	yes/no	Confirmation that the study was eligible to be included in the review
Extractor	yes/no	Name of data extractors
Extraction date	[Text]	Date of data extraction
Comment extra information	[Text]	what information was extracted
Search data	publication/registry	Specification wether the trial was published or not
Protocol or Statistical Analysis Plan available	yes/no	
Type of disease	Loiasis, mansonellosis, onchocerciasis	Microfilarial infection investigated
Trial registration number	[Text]	ID of registered trial protocol
Country	[Text]	Country where study was conducted
Primary objective	Efficacy, safety or both	
Number of arms	[Integer]	
Intervention	[Text]	Treatment used (excluding control)
Doses	[Text]	Dose used for treatment
Control	e.g. placebo/standard treatment/ no treatment	Type of control used
Control details	e.g. [Text]	What is the placebo or the standard drug
Method of randomization described	yes/no	Randomization method described in manuscript, protocol, or SAP
Type of randomization	e.g., simple randomization, block randomization, stratified randomization	
Strata	[Text]	If stratified randomization, what is the variable used for the stratification
Type of blinding	e.g., double-blind, single-blind, open label	Who was blinded
Type of the design	Superiority or non-inferiority	Clinical trial design
Sample size calculation	yes or no	Was a sample size calculation provided?
Information from author	yes or no	Was a sample size calculation provided by an author?
Sample size planned	[Integer]	The total number of pre-planned participants
Sample size in intervention group	[Integer]	Sample size after randomization (treatment group)
Sample size in placebo group	[Integer]	Sample size after randomization (control group)
Missing data primary endpoint	[Integer]	Randomized participants lost for primary endpoint data

Continued on next page

Table 1 – continued from previous page

Nr	Title of article/registry	Primary endpoint
Missing data follow-up	[Integer]	Randomized participants lost for the entire follow-up
Follow-up total	[Integer]	
Unit	day/month/year	
Primary endpoint identified	yes or no	Primary endpoint identified in manuscript or protocol
Type of primary outcome	Quantitative/qualitative	
Time point for the primary	[Integer]	
Maximum time point for the primary	[Integer]	The largest time point reported when several time points are considered
Unit primary endpoint	day/month/year	Unit of time
Detail of the primary outcome	[Text]	Specification of the primary outcome
Type of primary efficacy	Quantitative/qualitative	
Is microfilaria endpoint	yes/no	
Type from mf	[mf load/reduction]	Specification of the primary outcome when defined based on microfilaria
Primary objective	efficacy and/or safety	
Summary measure	Arithmetic mean, geometric mean, median, etc.	Summary used for the primary outcome
Phase of the RCT	[Integer]	
Primary analysis	[Text]	Statistical method for primary outcome
Interaction variables in the model	[Text]	Variables used for the interaction in the model
Analysis population	ITT or PP	Population used for decision making
Statistical method for missing data	[Text]	Approach used
Multiplicity (if applicable)	[Text]	Which adjustment for multiplicity was applied?
Interim analysis	yes/no	Were interim analyses conducted?
Significance alpha	[Integer]	Significance level used
Sideness of testing	1,2	One-sided or two-sided test
