# Supplementary material for: Design and analysis of randomized clinical trials for onchocerciasis, loiasis and mansonellosis: A systematic review
Source: PLoS Negl Trop Dis. 2026 Feb 20;20(2):e0013992. doi: 10.1371/journal.pntd.0013992 (PMC12952602; doi:10.1371/journal.pntd.0013992)
Supplement: S3 Table — (PDF) [file pntd.0013992.s003.pdf]

Nr	Title of article/registry	Primary endpoint
**Onchocerciasis**		
1	A randomized, open-label study of the tolerability and efficacy of one or three daily doses of ivermectin plus diethylcarbamazine and albendazole (IDA) versus one dose of ivermectin plus albendazole (IA) for treatment of onchocerciasis [1]	Compare rates and types of severe adverse and success fertility
2	Comparison of Repeated Doses of Ivermectin Versus Ivermectin Plus Albendazole for the Treatment of Onchocerciasis: A Randomized, Open-label, Clinical Trial [2]	Success in clearance
3	Single dose moxidectin versus ivermectin for *Onchocerca volvulus* infection in Ghana, Liberia, and the Democratic Republic of the Congo: a randomised, controlled, double-blind phase 3 trial [3]	Skin mf density
4	A randomized, single-ascending-dose, ivermectin-controlled, double-blind study of moxidectin in **Onchocerca volvulus** infection [4]	Incidence of clinical adverse events and clinically significant laboratory test results
5	Macrofilaricidal activity after doxycycline only treatment of *Onchocerca volvulus* in an area of *Loa loa* co-endemicity: a randomized controlled trial [5]	Sustained amicrofilaridermia in doxycycline or doxycycline and IVM-treated patients compared with IVM treated-patients assessed by levels of microfilaridermia in skin biopsies
6	Effects of Ivermectin with and without doxycycline on clinical symptoms of onchocerciasis [6]	Improvement of clinical features
7	Doxycycline Leads to Sterility and Enhanced Killing of Female Onchocerca volvulus Worms in an Area With Persistent Microfilaridermia After Repeated Ivermectin Treatment: A Randomized, Placebo-Controlled, Double-Blind Trial [7]	Proportion of sterile or dead female *O. volvulus* worms in nodules from doxycycline-treated patients 20 months after start of drug administration
8	The co-administration of ivermectin and albendazole–safety, pharmacokinetics and efficacy against *Onchocerca volvulus* [8]	Death or sterilization
9	Comparison of Doxycycline, Minocycline, Doxycycline plus Albendazole and Albendazole Alone in Their Efficacy against Onchocerciasis in a Randomized, Open-Label, Pilot Trial [9]	Absence of Wolbachia endobacteria in adult worms assessed by immunohistology
10	Effects of standard and high doses of ivermectin on adult worms of *Onchocerca volvulus*: a randomised controlled trial [10]	Vital status of female worms assessed from proportion and mean number of female worms in the MD category per nodule
11	Wolbachia endobacteria depletion by doxycycline as antifilarial therapy has macrofilaricidal activity in onchocerciasis:a randomized placebo-controlled study [11]	Presence of mf in the skin of the patients
12	Retarded Onchocerca volvulus L1 to L3 larval development in the Simulium damnosum vector after anti-wolbachial treatment of the human host [12]	Difference between follow-ups and pre-treatment regarding % of L3-larvae to account for development between pre- and after treatment in each patient
13	No depletion of Wolbachia from *Onchocerca volvulus* after a short course of rifampin and/or azithromycin [13]	-
14	Comparison of doxycycline alone vs doxycycline plus rifampicin in their efficacy against onchocerciasis [9]	Rates of nodules
15	Safety and Efficacy of Novel Combination Regimens for Treatment of Onchocerciasis [14]	Rates and types of severe or serious adverse events, proportion of all adult female worms that are fertile after 24 months
16	Study to Assess Adverse Events, Change in Disease Activity and How Oral ABBV-4083 Capsules When Given Alone or In Combination With Albendazole Capsules Moves in The Body of Adult Participants With *Onchocerca Volvulus* Infection [15]	-

Continued on next page

Table 1 – continued from previous page

Nr	Title of article/registry	Primary endpoint
17	Efficacy of Ivermectin and Albendazole Against Onchocerciasis in the Volta Region, Ghana [16]	To compare parasitologic efficacy measured by % fertile adult female worms in nodules at 36 months after administration of annual doses of oral IVM alone versus IVM plus ALB given annually or biannually
18	Emodepside Phase II Trial for Treatment of Onchocerciasis [17]	Absence of live female adult worms with normal embryogenesis assessed by histological examination of nodules; Absence of skin mf across four skin snips; Absence of skin mf assessed across all skin snips
19	The efficacy of Rifapentine plus Moxifloxacin against Onchocerciasis. [18]	Absence of Wolbachia endobacteria in female adult worms
20	Exploratory efficacy assessment of Rifampicin and ALB to treat Onchocerciasis in areas of co-endemicity with Loiasis [19]	Evaluation of adult female worm embryogenesis assessed by immunohistology 18 (+3) months after treatment onset (normal, degenerated or no embryos)
21	Safety and Efficacy of Annual or Biannual Doses of Mox or IVM for Onchocerciasis [20]	Proportion of moxidectin annual and biannual recipients with sustained mf response at month 12
22	The efficacy of Rifampicin plus Albendazole against Lymphatic filariasis and Onchocerciaisis [21]	Proportion of dead adult worms and MF assessed by immunohistology 20 months after treatment onset (where day 0 is start of drug administration), Absence of Wolbachia endobacteria in adult female worms assessed by immunohistology 20 months after treatment onset
23	A clinical trial to investigate efficacy and safety of oxfendazole compared with placebo in adults with trichuriasis, and /or mansonellosis, and/or onchocerciasis and/or loiasis [22]	parasite load at month 12 (mf count)
**Loiasis**		
24	Safety and Efficacy of Levamisole in Loiasis: A Randomized, Placebo-controlled, Double-blind Clinical Trial [23]	Absence of severe adverse events during the first week
25	Effect of Two or Six Doses 800 mg of Albendazole (ALB) Every Two Months on *Loa loa* Microfilaraemia: A Double Blind, Randomized, Placebo-Controlled Trial [24]	Proportion with 50% reduced mf
26	Efficacy, safety, and tolerability of albendazole and ivermectin based regimens for the treatment of microfilaraemic loiasis in adult patients in Gabon: A randomized controlled assessor blinded clinical trial [25]	Proportion with mf below 100 mf/ml
27	Effects of a 3-day regimen of albendazole (800 mg daily) on *Loa loa* microfilaraemia [26]	Mf count
28	A controlled trial to assess the effect of quinine, chloroquine, amodiaquine, and artesunate on *Loa loa* microfilaremia [27]	Mf count
29	Randomized, controlled, double-blind trial with ivermectin on *Loa loa* microfilaraemia: efficacy of a low dose (approximately 25 microg/kg) versus current standard dose (150 microg/kg) [28]	Mf count
30	Human loiasis in a Cameroonian village: a double-blind, placebo-controlled, crossover clinical trial of a three-day albendazole regimen [29]	Mf count

Continued on next page

Table 1 – continued from previous page

Nr	Title of article/registry	Primary endpoint
31	Post-treatment Effects of Ivermectin (IVM) or Diethylcarbamazine (DEC) in Loiasis [30]	Peak % of Baseline Eosinophil Count Measured 7 Days Post-treatment
32	A Randomized, Placebo-controlled, Double-blind Pilot Study of Single-dose Humanized Anti-IL5 Antibody (Reslizumab) for the Reduction of Eosinophilia Following Diethylcarbamazine Treatment of *Loa loa* Infection [31]	Reduction in absolute eosinophil count during the first week of DEC treatment
33	A 3- to 5-day Clinical Trial of Levamisole in Loiasis Infected Subjects [32]	Proportion of adverse event
34	Efficacy and Microfilaricidal Kinetics of Imatinib for the Treatment of *Loa Loa* [33]	Percent of baseline *Loa.* mf
35	Clinical Trial Evaluating the Safety and Efficacy of Moxidectin 2 mg Ivermectin-controlled in *Loa Loa* Microfilaremic Patients [34]	Absence of severe adverse events
36	21-day Double-blind Trial of Albendazole in Adults With *Loa Loa* Microfilaremia [32]	Tolerance of ALB 400mg/day
37	A clinical phase IIa randomized, ascending dose, placebo-controlled, assessor-blind, safety, tolerability and efficacy study of orally administered Moxidectin in subjects with microfilaraemic *Loa loa* infection [35]	The primary purpose of this study is to determine the safety and tolerability of single dose 8 mg moxidectin (MOX) in subjects infected with L. loa compared to ivermectin (IVM)
38	A clinical trial to investigate efficacy and safety of oxfendazole compared with placebo in adults with trichuriasis [22]	parasite load at month 12 (mf count)
39	A randomized, dose escalation, phase II, proof of concept trial evaluating the activity of oxfendazole for the treatment of loiasis [36]	The primary efficacy outcome is Safety and Tolerability of OXF in patients treated with loiasis during first 28 days post treatment for each dose level in the Intention To Treat Population (ITT)
**Mansonellosis**		
40	Phase III Clinical Trial to Evaluate Ivermectin in the Reduction of *Mansonella ozzardi* infection in the Brazilian Amazon [37]	Mf count
41	The Efficacy of Doxycycline Treatment on *Mansonella perstans* Infection: An Open-Label, Randomized Trial in Ghana [38]	Assessment of the mf load
42	A randomized trial of doxycycline for *Mansonella perstans* infection [39]	Proportion of mf reduction >50% or not
43	A randomised, double-blind field trial of ivermectin alone and in combination with albendazole for the treatment of *Mansonella perstans* infections in Uganda [40]	Mf periodicity patterns
44	A clinical trial to investigate efficacy and safety of oxfendazole compared with placebo in adults with trichuriasis [22]	parasite load at month 12 (mf count)

References

1. Opoku NO, Doe F, Dubben B, Fetcho N, Fischer K, Fischer PU, et al. A randomized, open-label study of the tolerability and efficacy of one or three daily doses of ivermectin plus diethylcarbamazine and albendazole (IDA) versus one dose of ivermectin plus albendazole (IA) for treatment of onchocerciasis. PLoS Neglected Tropical Diseases. 2023 May;17(5):e0011365. Doi:10.1371/journal.pntd.0011365.

2. Batsa Debrah L, Klarmann-Schulz U, Osei-Mensah J, Dubben B, Fischer K, Mubarik Y, et al. Comparison of Repeated Doses of Ivermectin Versus Ivermectin Plus Albendazole for the Treatment of Onchocerciasis: A

Randomized, Open-label, Clinical Trial. Clinical Infectious Diseases. 2020 Aug;71(4):933–943. Doi:10.1093/cid/ciz889.

3. Opoku NO, Bakajika DK, Kanza EM, Howard H, Mambandu GL, Nyathirombo A, et al. Single dose moxidectin versus ivermectin for Onchocerca volvulus infection in Ghana, Liberia, and the Democratic Republic of the Congo: a randomised, controlled, double-blind phase 3 trial. Lancet. 2018;392(10154):1207–1216. Doi:10.1016/S0140-6736(17)32844-1.

4. Awadzi K, Opoku NO, Attah SK, Lazdins-Helds J, Kuesel AC. A randomized, single-ascending-dose, ivermectin-controlled, double-blind study of moxidectin in Onchocerca volvulus infection. PLoS Neglected Tropical Diseases. 2014 Jun;8(6):e2953. Doi:10.1371/journal.pntd.0002953.

5. Turner JD, Tendongfor N, Esum M, Johnston KL, Langley RS, Ford L, et al. Macrofilaricidal Activity after Doxycycline Only Treatment of Onchocerca volvulus in an Area of Loa loa Co-Endemicity: A Randomized Controlled Trial. PLoS Neglected Tropical Diseases. 2010 Apr;4(4):e660. Doi:10.1371/journal.pntd.0000660.

6. Masud H, Qureshi TQ, Dukley M. Effects of Ivermectin with and without doxycycline on clinical symptoms of onchocerciasis. Journal of the College of Physicians and Surgeons–Pakistan: JCPSP. 2009 Jan;19(1):34–38. PMID: 19149978.

7. Debrah AY, Specht S, Klarmann-Schulz U, Batsa L, Mand S, Marfo-Debrekyei Y, et al. Doxycycline Leads to Sterility and Enhanced Killing of Female Onchocerca volvulus Worms in an Area With Persistent Microfilaridermia After Repeated Ivermectin Treatment: A Randomized, Placebo-Controlled, Double-Blind Trial. Clinical Infectious Diseases. 2015 Aug;61(4):517–526. Doi:10.1093/cid/civ363.

8. Awadzi K, Edwards G, Duke BOL, Opoku NO, Attah SK, Addy ET, et al. The co-administration of ivermectin and albendazole–safety, pharmacokinetics and efficacy against Onchocerca volvulus. Annals of Tropical Medicine and Parasitology. 2003 Mar;97(2):165–178. Doi:10.1179/000349803235001697.

9. Klarmann-Schulz U, Specht S, Debrah AY, Batsa L, Ayisi-Boateng NK, Osei-Mensah J, et al. Comparison of Doxycycline, Minocycline, Doxycycline plus Albendazole and Albendazole Alone in Their Efficacy against Onchocerciasis in a Randomized, Open-Label, Pilot Trial. PLoS neglected tropical diseases. 2017 Jan;11(1):e0005156. Doi:10.1371/journal.pntd.0005156.

10. Gardon J, Boussinesq M, Kamgno J, Gardon-Wendel N, Demanga-Ngangue n, Duke BOL. Effects of standard and high doses of ivermectin on adult worms of Onchocerca volvulus: a randomised controlled trial. Lancet. 2002 Jul;360(9328):203–210. Doi:/10.1016/S0140-6736(02)09456-4.

11. Hoerauf A, Specht S, Büttner M, Pfarr K, Mand S, Fimmers R, et al. Wolbachia endobacteria depletion by doxycycline as antifilarial therapy has macrofilaricidal activity in onchocerciasis: a randomized placebo-controlled study. Medical Microbiology and Immunology. 2008 Sep;197(3):295–311. Doi:10.1007/s00430-007-0062-1.

12. Albers A, Esum ME, Tendongfor N, Enyong P, Klarmann U, Wanji S, et al. Retarded Onchocerca volvulus L1 to L3 larval development in the Simulium

damnosum vector after anti-wolbachial treatment of the human host. Parasites & Vectors. 2012 Jan;5(1). Doi:10.1186/1756-3305-5-12.

13. Richards FO, Amann J, Arana B, Punkosdy G, Klein R, Blanco C, et al. No depletion of Wolbachia from Onchocerca volvulus after a short course of rifampin and/or azithromycin. The American Journal of Tropical Medicine and Hygiene. 2007 Nov;77(5):878–882. Doi:10.4269/ajtmh.2007.77.878.

14. Safety and Efficacy of Novel Combination Regimens for Treatment of Onchocerciasis. clinicaltrials.gov; 2024. NCT06070116.

15. A Phase-II, Randomised, Double-blind, Parallel-group, Proof-of-concept Trial to Investigate ABBV-4083 Given for 7 or 14 Days or in Combination With Albendazole in Subjects With Onchocerca Volvulus Infection, Comprising: Part 1 to Investigate Safety, Tolerability, Efficacy for Dose-Ranging and Pharmacokinetics; Part 2 to Investigate Efficacy of Selected Doses, Safety, Tolerability and Pharmacokinetics. clinicaltrials.gov; 2024. NCT04913610.

16. Comparison of Ivermectin Alone With Albendazole (ALB) Plus Ivermectin (IVM) in Their Efficacy Against Onchocerciasis in the Volta Region, Ghana. clinicaltrials.gov; 2019. NCT02078024.

17. Emodepside Phase II Trial for Treatment of Onchocerciasis. clinicaltrials.gov; 2021. NCT05180461.

18. Klarmann-Schulz U, Debrah A. The efficacy of rifapentine plus moxifloxacin against onchocerciasis: a randomized, open label pilot trial.; 2014. ISRCTN43697583. Doi:10.1186/ISRCTN43697583.

19. Wanji S, Hoerauf A. Exploratory efficacy assessment of Rifampicin and Albendazole to treat Onchocerciasis in areas of co-endemicity with Loiasis; 2021. ISRCTN38954299. Doi:10.1186/ISRCTN38954299.

20. Ukety T. A Randomized, Double Blind, Parallel Trial in the Democratic Republic of Congo (DRC) Comparing the Safety and Efficacy of Annual or Biannual Doses of Moxidectin or Ivermectin for Treatment of Onchocerciasis. clinicaltrials.gov; 2024. NCT03876262.

21. Batsa AYJL. The efficacy of Rifampicin plus Albendazole against Lymphatic filariasis and Onchocerciaisis.; 2020. PACTR202009704006025.

22. A phase IIa, multi-country, randomized, placebo-controlled, double-blinded, adaptive, basket trial to assess the efficacy and safety of oxfendazole in adults with trichuriasis, and /or mansonellosis, and/or onchocerciasis and/or loiasis; 2024. PACTR202412611774752.

23. Campillo JT, Bikita P, Hemilembolo M, Louya F, Missamou F, Pion SDS, et al. Safety and Efficacy of Levamisole in Loiasis: A Randomized, Placebo-controlled, Double-blind Clinical Trial. Clinical Infectious Diseases. 2022 Aug;75(1):19–27. Doi:10.1093/cid/ciab906.

24. Kamgno J, Nguipdop-Djomo P, Gounoue R, Téjiokem M, Kuesel AC. Effect of Two or Six Doses 800 mg of Albendazole Every Two Months on Loa loa Microfilaraemia: A Double Blind, Randomized, Placebo-Controlled Trial. PLoS Neglected Tropical Diseases. 2016 Mar;10(3):e0004492. Doi:10.1371/journal.pntd.0004492.

25. Zoleko-Manego R, Kreuzmair R, Veletzky L, Ndzebe-Ndoumba W, Ekoka Mbassi D, Okwu DG, et al. Efficacy, safety, and tolerability of albendazole and ivermectin based regimens for the treatment of microfilaraemic loiasis in adult patients in Gabon: A randomized controlled assessor blinded clinical trial. PLoS neglected tropical diseases. 2023 Aug;17(8):e0011584. Doi:10.1371/journal.pntd.0011584.

26. Tsague-Dongmo L, Kamgno J, Pion SDS, Moyou-Somo R, Boussinesq M. Effects of a 3-day regimen of albendazole (800 mg daily) on Loa loa microfilaraemia. Annals of Tropical Medicine and Parasitology. 2002 Oct;96(7):707–715. Doi:10.1179/000349802125001933.

27. Kamgno J, Djomo PN, Pion SD, Thylefors B, Boussinesq M. A controlled trial to assess the effect of quinine, chloroquine, amodiaquine, and artesunate on Loa loa microfilaremia. The American Journal of Tropical Medicine and Hygiene. 2010 Mar;82(3):379–385. Doi:10.4269/ajtmh.2010.09-0573.

28. Kamgno J, Pion SDS, Tejiokem MC, Twum-Danso NAY, Thylefors B, Boussinesq M. Randomized, controlled, double-blind trial with ivermectin on Loa loa microfilaraemia: efficacy of a low dose (approximately 25 microg/kg) versus current standard dose (150 microg/kg). Transactions of the Royal Society of Tropical Medicine and Hygiene. 2007 Aug;101(8):777–785. Doi:10.1016/j.trstmh.2007.03.018.

29. Tabi TE, Befidi-Mengue R, Nutman TB, Horton J, Folefack A, Pensia E, et al. Human loiasis in a Cameroonian village: a double-blind, placebo-controlled, crossover clinical trial of a three-day albendazole regimen. The American Journal of Tropical Medicine and Hygiene. 2004 Aug;71(2):211–215. Doi:10.4269/ajtmh.2004.71.211.

30. Herrick JA, Legrand F, Gounoue R, Nchinda G, Montavon C, Bopda J, et al. Posttreatment Reactions After Single-Dose Diethylcarbamazine or Ivermectin in Subjects With Loa loa Infection. Clinical Infectious Diseases. 2017 Apr;64(8):1017–1025. Doi:10.1093/cid/cix016.

31. Legrand F, Herrick J, Makiya M, Ramanathan R, Thompson R, Rampertaap S, et al. A Randomized, Placebo-controlled, Double-blind Pilot Study of Single-dose Humanized Anti-IL5 Antibody (Reslizumab) for the Reduction of Eosinophilia Following Diethylcarbamazine Treatment of Loa loa Infection. Clinical Infectious Diseases. 2021 Oct;73(7):e1624–e1631. Doi:10.1093/cid/ciaa1365.

32. Randomized, Double-blind Trial Evaluating the Safety and Efficacy of a 3- or 5-Day Course of Levamisole 2.5 mg/kg in Subjects With Loa Loa Microfilaremia. Institut de Recherche pour le Developpement; 2024. NCT06252961.

33. A Double-blinded, Randomized, Placebo-Controlled Dose Escalation Study to Examine the Efficacy and Microfilaricidal Kinetics and Safety of Imatinib for the Treatment of Loa Loa (A Pilot Study). NIAID; 2022. NCT02644525.

34. Randomized Clinical Trial, Double-blind, Single-dose Drug and Escalating Infection Intensities, Evaluating the Safety and Efficacy of Moxidectin 2 Mg, Ivermectin-controlled, in Loa Loa Microfilaremic Patients; 2025. NCT04049851.

35. Mombo G. A clinical phase IIa randomized, ascending dose, placebo-controlled, assessor-blind, safety, tolerability and efficacy study of orally administered

Moxidectin in subjects with microfilaraemic Loa loa infection; 2023. PACTR202303704849277.

36. Adegnika A. A randomized, dose escalation, phase II, proof of concept trial evaluating the activity of oxfendazole for the treatment of loiasis; 2024. PACTR202411787280874.

37. de Almeida Basano S, de Souza Almeida Aranha Camargo J, Fontes G, Pereira AR, Medeiros JF, de Oliveira Laudisse MC, et al. Phase III Clinical Trial to Evaluate Ivermectin in the Reduction of Mansonella ozzardi infection in the Brazilian Amazon. The American Journal of Tropical Medicine and Hygiene. 2018 Mar;98(3):786–790. Doi:10.4269/ajtmh.17-0698.

38. Batsa Debrah L, Phillips RO, Pfarr K, Klarmann-Schulz U, Opoku VS, Nausch N, et al. The Efficacy of Doxycycline Treatment on Mansonella perstans Infection: An Open-Label, Randomized Trial in Ghana. The American Journal of Tropical Medicine and Hygiene. 2019 Jul;101(1):84–92. Doi:10.4269/ajtmh.18-0491.

39. Coulibaly Y, Dembele B, Diallo A, Lipner E, Doumbia S, Coulibaly S, et al. A randomized trial of doxycycline for Mansonella perstans infection. The New England journal of medicine. 2009 Aug;361(15). Doi:10.1056/NEJMoa0900863.

40. Asio SM, Simonsen PE, Onapa AW. A randomised, double-blind field trial of ivermectin alone and in combination with albendazole for the treatment of Mansonella perstans infections in Uganda. Transactions of The Royal Society of Tropical Medicine and Hygiene. 2009 Mar;103(3):274–279. Doi:10.1016/j.trstmh.2008.10.038.
