# Supplementary material for: Design and analysis of randomized clinical trials for onchocerciasis, loiasis and mansonellosis: A systematic review
Source: PLoS Negl Trop Dis. 2026 Feb 20;20(2):e0013992. doi: 10.1371/journal.pntd.0013992 (PMC12952602; doi:10.1371/journal.pntd.0013992)
Supplement: S4 Table — Information on lower and upper limits of mf counts (and corresponding mf units), lower and upper limits of age and weight (sex-specific, if specified) for the 44 trials. (PDF) [file pntd.0013992.s004.pdf]

Nr	Registration number	Mf lower	Mf upper	Unit mf	Age lower (year)	Age upper (year)	Weight lower (kg) male	Weight low (kg) female	Weight upper (kg) male and female
**Onchocerciasis**									
1	NCT04188301	3		Mf/mg	16	70	-	-	-
2	ISRCTN50035143	-	-	-	18	60	40	40	-
3	NCT00790998	10	-	mf/mg	12	-	30	30	-
4	NCT00300768	-	-	-	18	60	45	40	-
5	ISRCTN48118452	10	-	mf/mg	15	60	40	40	-
6	-	-	-	-	16	-	-	-	-
7	ISRCTN66649839	-	-	-	18	50	40	40	-
8	not found	15	-	mf/mg	19	54	-	-	-
9	ISRCTN06010453	-	-	mf/mg	18	55	40	40	-
10	-	10	114	mf/skin snip	18	60	-	-	-
11	ISRCTN71141922	10	-	mf/mg	18	62	40	40	-
12	-	10	-	Mf in skin biopsies	21	50	-	-	-
13	-	-	-	-	5	-	-	-	-
14	ISRCTN68861628	-	-	-	18	55	40	40	70
15	NCT06070116	-	-	mf/mg	18	75	-	-	-
16	NCT04913610	-	-	-	18	65	40	40	-
17	NCT02078024	5	-	mf/mg	18	60	40	40	-
18	NCT05180461	-	-	-	18	65	40	40	-
19	PACTR201608001754356 - ISRCTN43697583	-	-	-	18	55	45	45	-
20	ISRCTN38954299	-	-	-	18	55	50	50	90
21	NCT03876262	10	-	mf/mg skin	12	-	-	-	88
22	PACTR202009704006025	-	-	-	18	55	45	45	-
23	PACTR202412611774752	-	-	-	18	65	45	45	-
**Loiasis**									
24	NCT04049630	-	-	-	18	65	45	40	85
25	ISRCTN25831558	1500	-	mf/mL	18	65	-	-	-
26	PACTR201807197019027	5000	50000	mf/ml	18	80	-	-	-
27	-	100	-	mf/ml	10	70	-	-	-
28	-	200	-	mf/ml	15	-	-	-	-
29	-	100	15000	mf/ml	15	70	-	-	-
30	-	-	-	-	-	-	-	-	-
31	NCT01593722	20	2000	mf/mL	20	60	-	-	-
32	NCT01111305	0	5000	mf/mL	18	65	-	-	-
33	NCT06252961	-	-	-	18	65	45	40	90
34	NCT02644525	500	2500	MF/mL	18	65	-	-	-
35	NCT04049851	-	1000	mf/mL	18	65	45	45	85
36	NCT06613997	-	-	-	18	70	40	40	90
37	PACTR202303704849277	1	2000	mf/ml	18	65	40	40	-
38	PACTR202412611774752	100	8000	mf/ml	18	65	45	45	-
39	PACTR202411787280874	100	8000	mf/ml	18	65	-	-	-
**Mansonellosis**									
40	PACTRN12609000005257	-	-	-	4	61	-	-	-
41	NCT02281643	-	-	-	10	55	40	40	-
42	NCT00340691	-	-	-	14	65	40	40	-
43	NCT00215280	20	-	MF/100UL	5	-	-	-	-
44	PACTR202412611774752	-	-	-	18	65	45	45	-
