# Supplementary material for: Design and analysis of randomized clinical trials for onchocerciasis, loiasis and mansonellosis: A systematic review
Source: PLoS Negl Trop Dis. 2026 Feb 20;20(2):e0013992. doi: 10.1371/journal.pntd.0013992 (PMC12952602; doi:10.1371/journal.pntd.0013992)
Supplement: S5 Table — Abbreviations: Ivermectin (IVM), diethylcarbamazine (DEC), albendazole (ALB), moxidectin (MOX), doxycline (DOXY). (PDF) [file pntd.0013992.s005.pdf]

Nr	Primary endpoint	Intervention	Control details
**Onchocerciasis**			
1	Compare rates and types of severe adverse and success fertility [1]	IDA: IVM+DEC+ALB different schedules	IA: IVM + ALB
2	Success in clearance [2]	IVM, ALB, different schedules	IVM
3	Skin mf density [3]	MOX	IVM
4	Incidence of clinical adverse events and clinically significant laboratory test results [4]	MOX, different doses and schedules	IVM
5	Sustained amicrofilaridermia in doxycycline or doxycycline and IVM-treated patients compared with IVM treated-patients assessed by levels of microfilaridermia in skin biopsies [5]	IVM+DOXY, different schedules	Placebo
6	Improvement of clinical features [6]	IVM+DOXY	IVM
7	Proportion of sterile or dead female *O. volvulus* worms in nodules from doxycycline-treated patients 20 months after start of drug administration [7]	DOXY	Placebo
8	Death or sterilization [8]	ALB; ALB+IVM	IVM
9	Absence of Wolbachia endobacteria in adult worms assessed by immunohistology [9]	ALB+DOXY, different schedules and combinations	ALB
10	Vital status of female worms assessed from proportion and mean number of female worms in the MD category per nodule [10]	IVM, different schedules and doses	IVM
11	Presence of mf in the skin of the patients [11]	DOXY, different schedules	Placebo
12	Difference between follow-ups and pre-treatment regarding % of L3-larvae to account for development between pre- and after treatment in each patient [12]	DOXY	Placebo
13	- [13]	Rifampin, azithromycin and combination	Placebo: multivitamin
14	Rates of nodules [9]	Rifampacin, DOXY different schedules and combinations	Placebo
15	Rates and types of severe or serious adverse events, proportion of all adult female worms that are fertile after 24 months [14]	MOX, ALB, IVM and DEC, different combinations	IVM+ALB
16	- [15]	Tylamac, ALB, different schedules and combinations	placebo+ALB
17	To compare parasitologic efficacy measured by % fertile adult female worms in nodules at 36 months after administration of annual doses of oral IVM alone versus IVM plus ALB given annually or biannually [16]	IVM, ALB, different combinations and schedules	IVM
18	Absence of live female adult worms with normal embryogenesis assessed by histological examination of nodules; Absence of skin mf across four skin snips; Absence of skin mf assessed across all skin snip [17]	Emodepside, different doses and schedules	Placebo
19	Absence of Wolbachia endobacteria in female adult worms	Moxifloxacin, rifapentine, different schedules	No treatment, DOXY

Continued on next page

Table 1 – continued from previous page

Nr	Primary endpoint	Intervention	Control details
20	Evaluation of adult female worm embryogenesis assessed by immunohistology 18 (+3) months after treatment onset (normal, degenerated or no embryos) [18]	Rifampicin, ALB, different doses and schedules	IVM, DOXY, ALB
21	Proportion of moxidectin annual and biannual recipients with sustained mf response at month 12 [19]	IVM, MOX, different schedules	IVM
22	Proportion of dead adult worms and MF assessed by immunohistology 20 months after treatment onset, Absence of Wolbachia endobacteria in adult female worms [20]	Rifampicin, Zentel, different schedules	IVM
23	Mf count at month 12 [21]	Oxfendazole (OXF), different doses and schedules	Placebo
**Loiasis**			
24	Absence of severe adverse events during the first week [22]	Levamisole, different doses	Placebo
25	Proportion with 50% reduced mf [23]	ALB	Placebo
26	Proportion with mf below 100 mf/ml [24]	ALB, IVM, different combinations	Loretadine
27	Mf count [25]	ALB	Placebo: multivitamin
28	Mf count [26]	Quinine, chloroquine, amodiaquine, and artesunate	Placebo: iron-folic acid
29	Mf count [27]	IVM, different schedules	IVM
30	Mf count [28]	ALB, different schedules	Placebo
31	Peak % of Baseline Eosinophil Count Measured 7 Days Post-treatment [29]	DEC	IVM
32	Reduction in absolute eosinophil count during the first week of DEC treatment [30]	Reslizumab	Placebo
33	Proportion of adverse event [31]	Levamisole	Placebo
34	Percent of baseline mf [32]	Imatinib, different doses	Placebo: vitamin tablets
35	Absence of severe adverse events [33]	MOX	IVM
36	Tolerance [31]	ALB	Placebo
37	Safety and tolerability [34]	MOX	Placebo + IVM
38	Mf count at month 12 [21]	OXF, different doses and schedules	Placebo
39	- [35]	OXF, different doses and schedules	Placebo
**Mansonellosis**			
40	Mf count [36]	IVM	Placebo
41	Assessment of the mf load [37]	DOXY	No treatment - delayed treatment
42	Proportion of mf reduction >50% or not [38]	DOXY	No treatment
43	Mf periodicity patterns [39]	IVM+ALB	IVM
44	Mf count at month 12 [21]	OXF, different doses and schedules	Placebo

References

1. Opoku NO, Doe F, Dubben B, Fetcho N, Fischer K, Fischer PU, et al. A randomized, open-label study of the tolerability and efficacy of one or three daily doses of ivermectin plus diethylcarbamazine and albendazole (IDA) versus one dose of ivermectin plus albendazole (IA) for treatment of onchocerciasis. PLoS Neglected Tropical Diseases. 2023 May;17(5):e0011365. Doi:10.1371/journal.pntd.0011365.

2. Batsa Debrah L, Klarmann-Schulz U, Osei-Mensah J, Dubben B, Fischer K, Mubarik Y, et al. Comparison of Repeated Doses of Ivermectin Versus Ivermectin Plus Albendazole for the Treatment of Onchocerciasis: A Randomized, Open-label, Clinical Trial. Clinical Infectious Diseases. 2020 Aug;71(4):933–943. Doi:10.1093/cid/ciz889.

3. Opoku NO, Bakajika DK, Kanza EM, Howard H, Mambandu GL, Nyathirombo A, et al. Single dose moxidectin versus ivermectin for Onchocerca volvulus infection in Ghana, Liberia, and the Democratic Republic of the Congo: a randomised, controlled, double-blind phase 3 trial. Lancet. 2018;392(10154):1207–1216. Doi:10.1016/S0140-6736(17)32844-1.

4. Awadzi K, Opoku NO, Attah SK, Lazdins-Helds J, Kuesel AC. A randomized, single-ascending-dose, ivermectin-controlled, double-blind study of moxidectin in Onchocerca volvulus infection. PLoS Neglected Tropical Diseases. 2014 Jun;8(6):e2953. Doi:10.1371/journal.pntd.0002953.

5. Turner JD, Tendongfor N, Esum M, Johnston KL, Langley RS, Ford L, et al. Macrofilaricidal Activity after Doxycycline Only Treatment of Onchocerca volvulus in an Area of Loa loa Co-Endemicity: A Randomized Controlled Trial. PLoS Neglected Tropical Diseases. 2010 Apr;4(4):e660. Doi:10.1371/journal.pntd.0000660.

6. Masud H, Qureshi TQ, Dukley M. Effects of Ivermectin with and without doxycycline on clinical symptoms of onchocerciasis. Journal of the College of Physicians and Surgeons–Pakistan: JCPSP. 2009 Jan;19(1):34–38. PMID: 19149978.

7. Debrah AY, Specht S, Klarmann-Schulz U, Batsa L, Mand S, Marfo-Debrekyei Y, et al. Doxycycline Leads to Sterility and Enhanced Killing of Female Onchocerca volvulus Worms in an Area With Persistent Microfilaridermia After Repeated Ivermectin Treatment: A Randomized, Placebo-Controlled, Double-Blind Trial. Clinical Infectious Diseases. 2015 Aug;61(4):517–526. Doi:10.1093/cid/civ363.

8. Awadzi K, Edwards G, Duke BOL, Opoku NO, Attah SK, Addy ET, et al. The co-administration of ivermectin and albendazole–safety, pharmacokinetics and efficacy against Onchocerca volvulus. Annals of Tropical Medicine and Parasitology. 2003 Mar;97(2):165–178. Doi:10.1179/000349803235001697.

9. Klarmann-Schulz U, Specht S, Debrah AY, Batsa L, Ayisi-Boateng NK, Osei-Mensah J, et al. Comparison of Doxycycline, Minocycline, Doxycycline plus Albendazole and Albendazole Alone in Their Efficacy against Onchocerciasis in a Randomized, Open-Label, Pilot Trial. PLoS neglected tropical diseases. 2017 Jan;11(1):e0005156. Doi:10.1371/journal.pntd.0005156.

10. Gardon J, Boussinesq M, Kamgno J, Gardon-Wendel N, Demanga-Ngangue n, Duke BOL. Effects of standard and high doses of ivermectin on adult worms of Onchocerca volvulus: a randomised controlled trial. Lancet. 2002 Jul;360(9328):203–210. Doi:/10.1016/S0140-6736(02)09456-4.

11. Hoerauf A, Specht S, Büttner M, Pfarr K, Mand S, Fimmers R, et al. Wolbachia endobacteria depletion by doxycycline as antifilarial therapy has macrofilaricidal activity in onchocerciasis: a randomized placebo-controlled study. Medical Microbiology and Immunology. 2008 Sep;197(3):295–311. Doi:10.1007/s00430-007-0062-1.

12. Albers A, Esum ME, Tendongfor N, Enyong P, Klarmann U, Wanji S, et al. Retarded Onchocerca volvulus L1 to L3 larval development in the Simulium damnosum vector after anti-wolbachial treatment of the human host. Parasites & Vectors. 2012 Jan;5(1). Doi:10.1186/1756-3305-5-12.

13. Richards FO, Amann J, Arana B, Punkosdy G, Klein R, Blanco C, et al. No depletion of Wolbachia from Onchocerca volvulus after a short course of rifampin and/or azithromycin. The American Journal of Tropical Medicine and Hygiene. 2007 Nov;77(5):878–882. Doi:10.4269/ajtmh.2007.77.878.

14. Safety and Efficacy of Novel Combination Regimens for Treatment of Onchocerciasis. clinicaltrials.gov; 2024. NCT06070116.

15. A Phase-II, Randomised, Double-blind, Parallel-group, Proof-of-concept Trial to Investigate ABBV-4083 Given for 7 or 14 Days or in Combination With Albendazole in Subjects With Onchocerca Volvulus Infection, Comprising: Part 1 to Investigate Safety, Tolerability, Efficacy for Dose-Ranging and Pharmacokinetics; Part 2 to Investigate Efficacy of Selected Doses, Safety, Tolerability and Pharmacokinetics. clinicaltrials.gov; 2024. NCT04913610.

16. Comparison of Ivermectin Alone With Albendazole (ALB) Plus Ivermectin (IVM) in Their Efficacy Against Onchocerciasis in the Volta Region, Ghana. clinicaltrials.gov; 2019. NCT02078024.

17. Emodepside Phase II Trial for Treatment of Onchocerciasis. clinicaltrials.gov; 2021. NCT05180461.

18. Wanji S, Hoerauf A. Exploratory efficacy assessment of Rifampicin and Albendazole to treat Onchocerciasis in areas of co-endemicity with Loiasis; 2021. ISRCTN38954299. Doi:10.1186/ISRCTN38954299.

19. Ukety T. A Randomized, Double Blind, Parallel Trial in the Democratic Republic of Congo (DRC) Comparing the Safety and Efficacy of Annual or Biannual Doses of Moxidectin or Ivermectin for Treatment of Onchocerciasis. clinicaltrials.gov; 2024. NCT03876262.

20. Batsa AYJL. The efficacy of Rifampicin plus Albendazole against Lymphatic filariasis and Onchocerciaisis.; 2020. PACTR202009704006025.

21. A phase IIa, multi-country, randomized, placebo-controlled, double-blinded, adaptive, basket trial to assess the efficacy and safety of oxfendazole in adults with trichuriasis, and /or mansonellosis, and/or onchocerciasis and/or loiasis; 2024. PACTR202412611774752.

22. Campillo JT, Bikita P, Hemilembolo M, Louya F, Missamou F, Pion SDS, et al. Safety and Efficacy of Levamisole in Loiasis: A Randomized, Placebo-controlled, Double-blind Clinical Trial. Clinical Infectious Diseases. 2022 Aug;75(1):19–27. Doi:10.1093/cid/ciab906.

23. Kamgno J, Nguipdop-Djomo P, Gounoue R, Téjiokem M, Kuesel AC. Effect of Two or Six Doses 800 mg of Albendazole Every Two Months on Loa loa Microfilaraemia: A Double Blind, Randomized, Placebo-Controlled Trial. PLoS Neglected Tropical Diseases. 2016 Mar;10(3):e0004492. Doi:10.1371/journal.pntd.0004492.

24. Zoleko-Manego R, Kreuzmair R, Veletzky L, Ndzebe-Ndoumba W, Ekoka Mbassi D, Okwu DG, et al. Efficacy, safety, and tolerability of albendazole and ivermectin based regimens for the treatment of microfilaraemic loiasis in adult patients in Gabon: A randomized controlled assessor blinded clinical trial. PLoS neglected tropical diseases. 2023 Aug;17(8):e0011584. Doi:10.1371/journal.pntd.0011584.

25. Tsague-Dongmo L, Kamgno J, Pion SDS, Moyou-Somo R, Boussinesq M. Effects of a 3-day regimen of albendazole (800 mg daily) on Loa loa microfilaraemia. Annals of Tropical Medicine and Parasitology. 2002 Oct;96(7):707–715. Doi:10.1179/000349802125001933.

26. Kamgno J, Djomo PN, Pion SD, Thylefors B, Boussinesq M. A controlled trial to assess the effect of quinine, chloroquine, amodiaquine, and artesunate on Loa loa microfilaremia. The American Journal of Tropical Medicine and Hygiene. 2010 Mar;82(3):379–385. Doi:10.4269/ajtmh.2010.09-0573.

27. Kamgno J, Pion SDS, Tejiokem MC, Twum-Danso NAY, Thylefors B, Boussinesq M. Randomized, controlled, double-blind trial with ivermectin on Loa loa microfilaraemia: efficacy of a low dose (approximately 25 microg/kg) versus current standard dose (150 microg/kg). Transactions of the Royal Society of Tropical Medicine and Hygiene. 2007 Aug;101(8):777–785. Doi:10.1016/j.trstmh.2007.03.018.

28. Tabi TE, Befidi-Mengue R, Nutman TB, Horton J, Folefack A, Pensia E, et al. Human loiasis in a Cameroonian village: a double-blind, placebo-controlled, crossover clinical trial of a three-day albendazole regimen. The American Journal of Tropical Medicine and Hygiene. 2004 Aug;71(2):211–215. Doi:10.4269/ajtmh.2004.71.211.

29. Herrick JA, Legrand F, Gounoue R, Nchinda G, Montavon C, Bopda J, et al. Posttreatment Reactions After Single-Dose Diethylcarbamazine or Ivermectin in Subjects With Loa loa Infection. Clinical Infectious Diseases. 2017 Apr;64(8):1017–1025. Doi:10.1093/cid/cix016.

30. Legrand F, Herrick J, Makiya M, Ramanathan R, Thompson R, Rampertaap S, et al. A Randomized, Placebo-controlled, Double-blind Pilot Study of Single-dose Humanized Anti-IL5 Antibody (Reslizumab) for the Reduction of Eosinophilia Following Diethylcarbamazine Treatment of Loa loa Infection. Clinical Infectious Diseases. 2021 Oct;73(7):e1624–e1631. Doi:10.1093/cid/ciaa1365.

31. Randomized, Double-blind Trial Evaluating the Safety and Efficacy of a 3- or 5-Day Course of Levamisole 2.5 mg/kg in Subjects With Loa Loa Microfilaremia. Institut de Recherche pour le Developpement; 2024. NCT06252961.

32. A Double-blinded, Randomized, Placebo-Controlled Dose Escalation Study to Examine the Efficacy and Microfilaricidal Kinetics and Safety of Imatinib for the Treatment of Loa Loa (A Pilot Study). NIAID; 2022. NCT02644525.

33. Randomized Clinical Trial, Double-blind, Single-dose Drug and Escalating Infection Intensities, Evaluating the Safety and Efficacy of Moxidectin 2 Mg, Ivermectin-controlled, in Loa Loa Microfilaremic Patients; 2025. NCT04049851.

34. Mombo G. A clinical phase IIa randomized, ascending dose, placebo-controlled, assessor-blind, safety, tolerability and efficacy study of orally administered Moxidectin in subjects with microfilaraemic Loa loa infection; 2023. PACTR202303704849277.

35. Adegnika A. A randomized, dose escalation, phase II, proof of concept trial evaluating the activity of oxfendazole for the treatment of loiasis; 2024. PACTR202411787280874.

36. de Almeida Basano S, de Souza Almeida Aranha Camargo J, Fontes G, Pereira AR, Medeiros JF, de Oliveira Laudisse MC, et al. Phase III Clinical Trial to Evaluate Ivermectin in the Reduction of Mansonella ozzardi infection in the Brazilian Amazon. The American Journal of Tropical Medicine and Hygiene. 2018 Mar;98(3):786–790. Doi:10.4269/ajtmh.17-0698.

37. Batsa Debrah L, Phillips RO, Pfarr K, Klarmann-Schulz U, Opoku VS, Nausch N, et al. The Efficacy of Doxycycline Treatment on Mansonella perstans Infection: An Open-Label, Randomized Trial in Ghana. The American Journal of Tropical Medicine and Hygiene. 2019 Jul;101(1):84–92. Doi:10.4269/ajtmh.18-0491.

38. Coulibaly Y, Dembele B, Diallo A, Lipner E, Doumbia S, Coulibaly S, et al. A randomized trial of doxycycline for Mansonella perstans infection. The New England journal of medicine. 2009 Aug;361(15). Doi:10.1056/NEJMoa0900863.

39. Asio SM, Simonsen PE, Onapa AW. A randomised, double-blind field trial of ivermectin alone and in combination with albendazole for the treatment of Mansonella perstans infections in Uganda. Transactions of The Royal Society of Tropical Medicine and Hygiene. 2009 Mar;103(3):274–279. Doi:10.1016/j.trstmh.2008.10.038.
