# Supplementary material for: Design and analysis of randomized clinical trials for onchocerciasis, loiasis and mansonellosis: A systematic review
Source: PLoS Negl Trop Dis. 2026 Feb 20;20(2):e0013992. doi: 10.1371/journal.pntd.0013992 (PMC12952602; doi:10.1371/journal.pntd.0013992)
Supplement: S6 Table — Frequencies are shown for two time periods 2000-2012 and 2013-2024. (PDF) [file pntd.0013992.s006.pdf]

Characteristic	Overall<br>N = 44	Onchocerciasis<br>N = 23	Loiasis<br>N = 16	Mansonellosis<br>N = 5
**Time periods 2000-2012**				
	n = 14	n = 8	n = 4	n = 2
**Control group**				
No treatment	2 (14%)	0 (0%)	1 (25%)	1 (50%)
Placebo	7 (50%)	5 (63%)	2 (50%)	0 (0%)
Standard drug/dose	5 (36%)	3 (38%)	1 (25%)	1 (50%)
**Method of randomization**				
Simple randomization	1 (7.1%)	1 (13%)	0 (0%)	0 (0%)
Block randomization	0 (0%)	0 (0%)	0 (0%)	0 (0%)
Stratified randomization	5 (36%)	2 (25%)	3 (75%)	0 (0%)
Not specified	8 (57%)	5 (63%)	1 (25%)	2 (100%)
**Time periods 2013-2024**				
	n = 30	n = 15	n = 12	n = 3
**Control group**				
No treatment	-	-	-	-
Placebo	13 (43%)	3 (20%)	8 (67%)	2 (67%)
Standard drug/dose	17 (57%)	12 (80%)	4 (33%)	1 (33%)
**Method of randomization**				
Simple randomization	2 (6.7%)	1 (6.7%)	1 (8.3%)	0 (0%)
Block randomization	7 (23%)	1 (6.7%)	5 (42%)	1 (33%)
Stratified randomization	9 (30%)	7 (47%)	2 (17%)	0 (0%)
Not specified	12 (40%)	6 (40%)	4 (33%)	2 (67%)
