# Supplementary material for: Design and analysis of randomized clinical trials for onchocerciasis, loiasis and mansonellosis: A systematic review
Source: PLoS Negl Trop Dis. 2026 Feb 20;20(2):e0013992. doi: 10.1371/journal.pntd.0013992 (PMC12952602; doi:10.1371/journal.pntd.0013992)
Supplement: S7 Table — Frequencies are shown for two time periods 2000-2012 and 2013-2024. (PDF) [file pntd.0013992.s007.pdf]

	Overall<br>N = 44	Onchocerciasis<br>N = 23	Loiasis<br>N = 16	Mansonellosis<br>N = 5
**Time periods 2000-2012**				
	n = 14	n = 8	n = 4	n = 2
**Efficacy endpoint**				
Qualitative	4 (29%)	3 (38%)	0 (0%)	1 (50%)
Quantitative	8 (57%)	3 (38%)	4 (100%)	1 (50%)
Both	0 (0%)	0 (0%)	0 (0%)	0 (0%)
Not available/not clear	2 (14%)	2 (25%)	0 (0%)	0 (0%)
**Primary efficacy**				
Qualitative	4 (29%)	3 (38%)	0 (0%)	1 (50%)
Quantitative	3 (21%)	2 (25%)	0 (0%)	1 (50%)
Both	0 (0%)	0 (0%)	0 (0%)	0 (0%)
Not available/not clear	0 (0%)	0 (0%)	0 (0%)	0 (0%)
**Primary efficacy microfilaria**				
Qualitative	2 (14%)	1 (12%)	0 (0%)	1 (50%)
Quantitative	2 (14%)	1 (12%)	0 (0%)	1 (50%)
Both	0 (0%)	0 (0%)	0 (0%)	0 (0%)
Not available/not clear	0 (0%)	0 (0%)	0 (0%)	0 (0%)
**Time periods 2013-2024**				
	n = 30	n = 15	n = 12	n = 3
**Efficacy endpoint**				
Qualitative	13 (43%)	9 (60%)	4 (33%)	0 (0%)
Quantitative	12 (40%)	5 (33%)	4 (33%)	3 (100%)
Both	2 (6.7%)	0 (0%)	2 (17%)	0 (0%)
Not available/not clear	2 (6.7%)	1 (7%)	1 (8.3%)	0 (0%)
**Primary efficacy**				
Qualitative	11 (37%)	9 (60%)	2 (17%)	0 (0%)
Quantitative	11 (37%)	4 (27%)	4 (33%)	3 (100%)
Both	1 (3%)	0 (0%)	1 (7%)	0 (0%)
Not available/not clear	1 (3%)	1 (7%)	0 (0%)	0 (0%)
**Primary efficacy mf**				
Qualitative	4 (13%)	2 (7%)	2 (13%)	0 (0%)
Quantitative	8 (27%)	3 (20%)	2 (17%)	3 (100%)
Both	1 (3%)	0 (0%)	1 (8%)	0 (0%)
Not available/not clear	0 (0%)	0 (0%)	0 (0%)	0 (0%)
