# Supplementary figures and images for: Design and analysis of randomized clinical trials for onchocerciasis, loiasis and mansonellosis: A systematic review
Source: PLoS Negl Trop Dis. 2026 Feb 20;20(2):e0013992. doi: 10.1371/journal.pntd.0013992 (PMC12952602; doi:10.1371/journal.pntd.0013992)

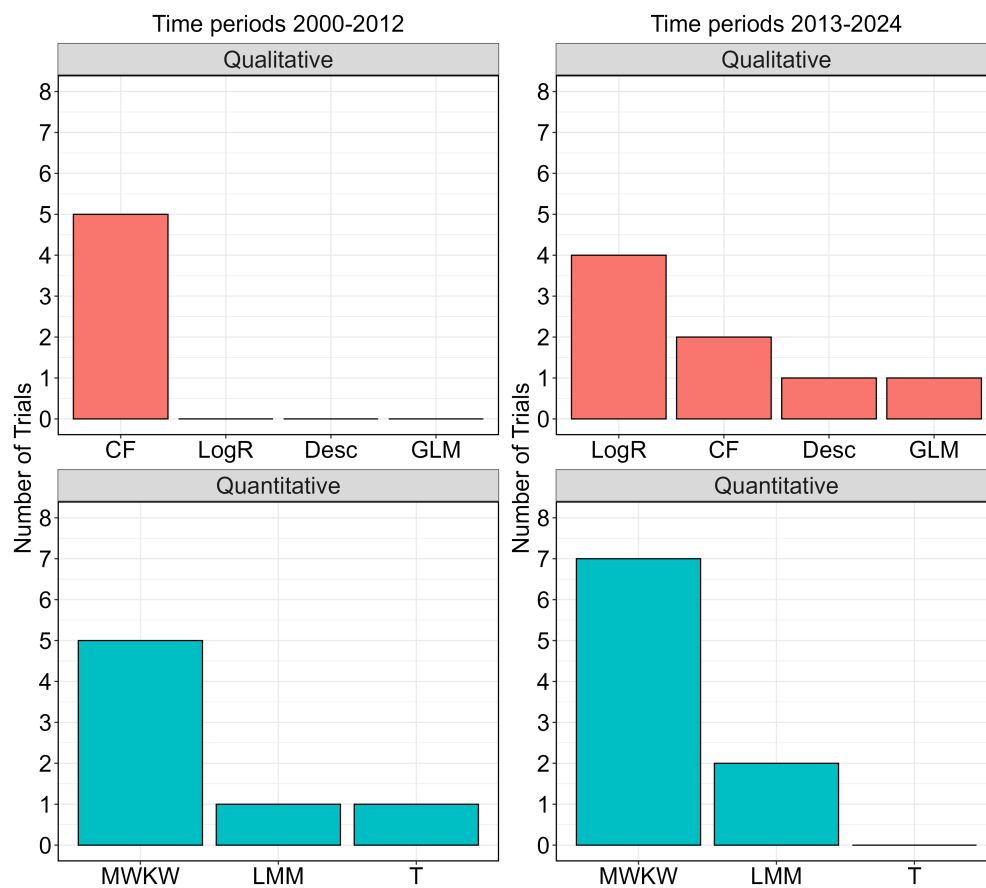

Supplement: S1 Fig — The quantitative methods are the non-parametric Mann-Whitney U or Kruskal-Wallis test (MWKW), the parametric t-test (TA), linear mixed model or repeated measures analysis of variance (LMM) and the Poisson regression (PR). The qualitative methods are descriptively only (Desc), chi-squared or Fisher’s exact test (CF), logistic regression (LogR), and generalized linear mixed model (GLMM). (PDF) [file pntd.0013992.s010.pdf]

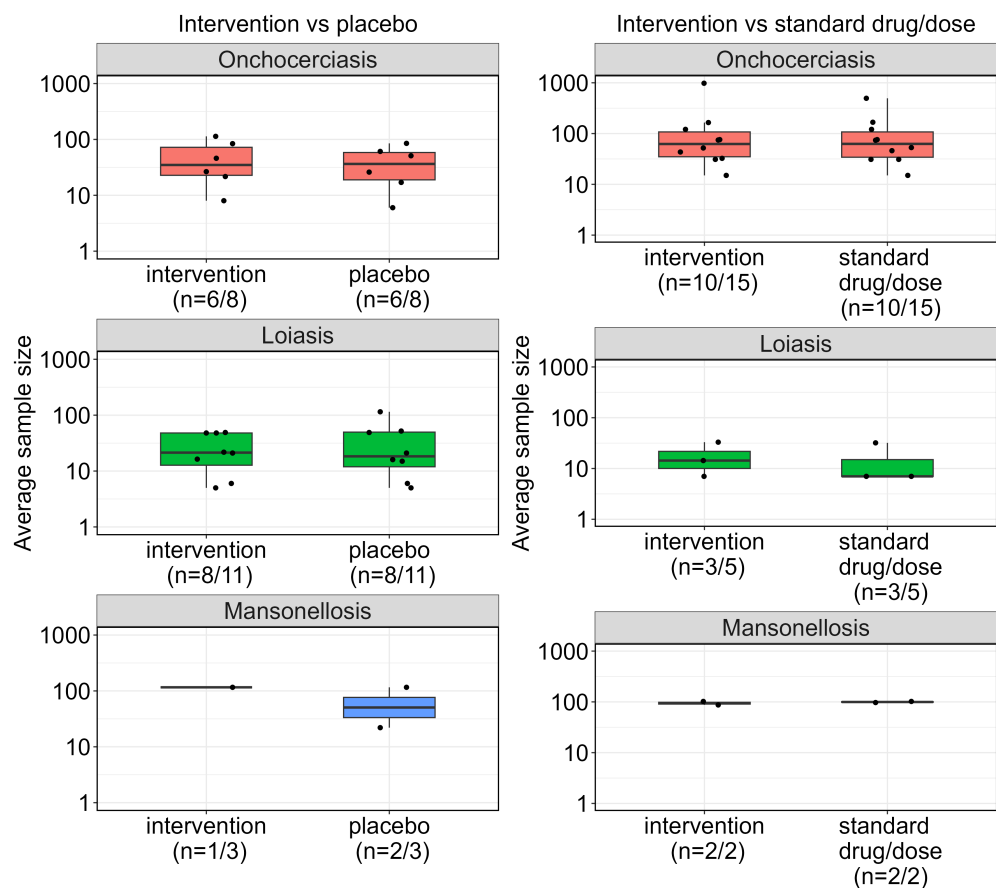

Supplement: S2 Fig — Note: The whiskers extend from the box to the minimum and maximum values excluding outliers. Outliers, defined as 1.5 times the interquartile range away from the box, are represented by points only. (PDF) [file pntd.0013992.s011.pdf]

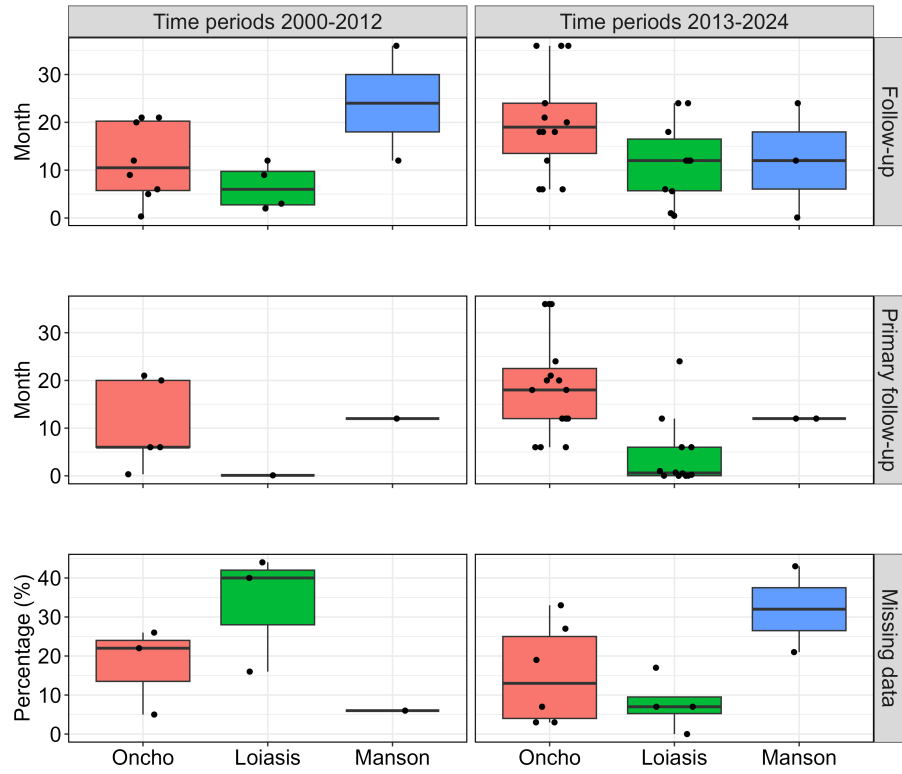

Supplement: S3 Fig — Values are shown for two time periods 2000-2012 and 2013-2024. (PDF) [file pntd.0013992.s012.pdf]

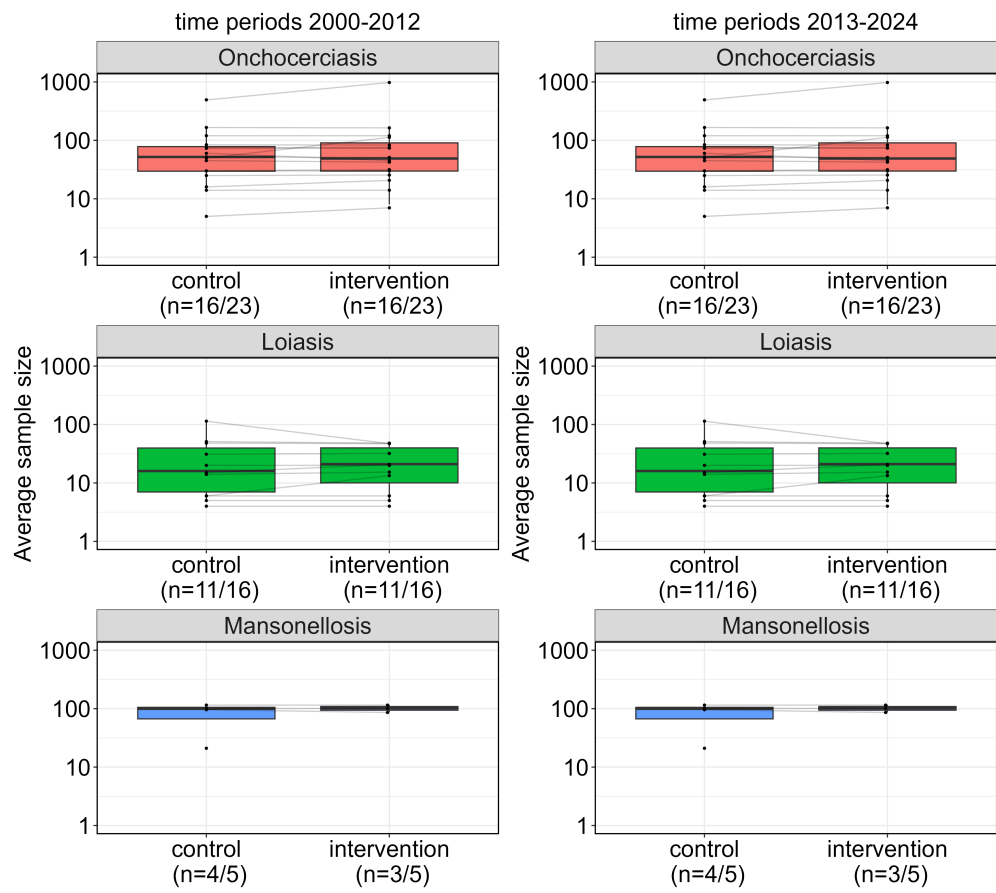

Supplement: S4 Fig — Log-transformed values are shown for two time periods 2000-2012 and 2013-2024. (PDF) [file pntd.0013992.s013.pdf]
