# Supplementary material for: Design and analysis of randomized clinical trials for onchocerciasis, loiasis and mansonellosis: A systematic review
Source: PLoS Negl Trop Dis. 2026 Feb 20;20(2):e0013992. doi: 10.1371/journal.pntd.0013992 (PMC12952602; doi:10.1371/journal.pntd.0013992)
Supplement: S1 Text — (PDF) [file pntd.0013992.s014.pdf]

# Protocol for systematic review

# V2.0

Fabrice Lotola Mougeni,

Marta Bofill Roig

Martin Posch

Sonja Zehetmayer

# Table of contents

**Introduction** ........ 2
Background ........ 3
Rational ........ 4
Research question ........ 3
Objectives ........ 4
**Method** ........ 4
Search strategy ........ 4
Eligibility criteria ........ 5
Intervention ........ 5
Data collection ........ 6
Variable extraction ........ 6

# Introduction

## Background

Loa loa, Mansonella and Onchocerca V. are microfilaria diseases that pose challenge in Sub-Saharan countries. To reduce their burden in this area, several drugs are used aiming to clear microfilaria density: IVM, DIOX, Mox, ALB, etc.... Statistical methods play an important role in the drug development process contributing in shift from medicine grounded in subjective opinions to a practice rooted in evidence-based approaches. This practice of evidence-based approach uses data collected to obtain meaningful results thanks to the recent development of biostatistical techniques to obtain better estimate of the treatment effect and reduce bias and thus contribute in guaranteeing the reliability and the validity of a clinical trials. These technics are applied differently depending on the type of primary endpoint or the focus of the clinical trials. Understanding several other parameters related to the clinical trials and the statistical methodology can help in designing a successful trial by reducing the type I error and increasing the probability of detecting an existing effect. This can also avoid poor interpretation, wrong conclusions, and maximize good clinical decision making. As far as we know, there is limited knowledge regarding a review of all the statistical methodology aspects related to the clinical trials focusing on these microfilaria diseases. This review focused on describing which statistical methodology are mostly used in such clinical trials. This ranges from the parameters characterizing the clinical trials focused on clearing microfilaria after treatment, the distribution of some parameters and their uncertainty, the design in terms of parameters to consider before planning the trial such as primary endpoint, the inclusion and exclusion criteria, the accrual rate, the dropout rate, to the statistical methods applied to deal with the estimation of the effect and dealing with the challenge of distribution.

### Research question

What is the characteristic of elements involved in a clinical trial and related to the statistical aspects of clinical trials on the microfilaria diseases: loiasis, mansonellosis, onchocerciasis. What is the current practice in statistical methodologies related to the clinical trials for these microfilariae to establish either the effect or the efficacy of drugs?

### Rational

Having an insight in the parameters involved in the planification of clinical trial and related to the statistical methodology aspect can contribute to improve the overall success of a trial by providing more overview on some specific parameters. It can also help in providing more familiar and suitable approach helping to estimate the statistical level encountered when analysing this kind of RCT data. Furthermore, it can contribute in identifying the most robust and reliable statistical approaches. This can also improve the recommendation in analysis plan in the same circumstance, or reveal limitation in statistical methods and seek how to promote simple and powerful method not used and excite extensive investigation on those methods used.

### Objectives

- To have an insight in the parameters characterizing the primary endpoint or the clinical trials focusing on microfilaria diseases.
- To describe or summarize the current knowledge on the statistical methods used for in clinical trials focused on the considered microfilaria diseases
- To identify how some factors related to the design of a clinical trials and the statistical methodology are often presented to serve as a contribution in providing a standardized guideline in statistical plan analysis.
- To understanding the current landscape of statistical methodology in clinical trials

## Method

To conduct this review, PRISMA – 2020 guideline for items to include was considered.

### Search strategy

The main research library is PubMed. Three other databases will be also considered: clinicaltrials.gov, trial search WHO, and the European registry. The search will be limited to human Randomized Clinical trial (RCTs) from January 1, 2000 to December 31, 2024 in English.

In Pubmed, we will use the following query for each disease:

- *Loa Loa*: (”loiasis”[MeSH Terms] OR ”loiasis”[All Fields] OR ”Loa loa”[All Fields]) AND ((randomizedcontrolledtrial[Filter]) AND (2000:2024[pdat]))
- Mansonella P.: (”mansonella”[MeSH Terms] OR ”mansonella”[All Fields] OR ”mansonelliasis”[MeSH Terms] OR ”mansonelliasis”[All Fields] OR ”mansonelliases”[All Fields]) AND ((randomizedcontrolledtrial[Filter]) AND (2000:2024[pdat]))

- Oncho V.: ”onchocerciasis”[MeSH Terms] OR ”onchocerciasis”[All Fields] OR ”onchocerciases”[All Fields] OR ”onchocerciasis”[MeSH Terms] OR ”onchocerciasis”[All Fields]) AND ((randomizedcontrolledtrial[Filter]) AND (2000:2024[pdat]))

Other registries:

- Loiasis OR Loa Loa OR Loiases
- mansonella OR mansonelliasis OR mansonelliases
- onchocerciasis OR onchocerciases OR onchocerciasis

**Eligibility criteria**

The following points were considered as selection criteria:

*Inclusion criteria*:

- Must be an RCT focused on efficacy or the effect of some drugs, or safety, focused on primary endpoint.
- Intervention must be specified, and
- The paper from peer reviewed journal.
- Must be between January 2000 and December 2024.

*Exclusion criteria*:

- inclusion criteria not met,
- paper discussing new methods instead of specific clinical trials,
- Phase I study
- Short report
- other type of RCTs focusing on other disease than the microfilaria: Loa loa, Onch V. and Mansonella.

**Intervention**

All the drugs used to treat microfilaria diseases between the considered period: IVM, ALB, and DIOX,...LEV, and so forth

**Data collection**

Spreadsheet containing some details about all the articles will be used as a data collection tool. Consistency between independent reviewers will be evaluated either using agreement statistics to estimate errors or simply consensus review for the discrepancy. Study flow diagram showing excluded and included articles and reasons will be presented.

**Classification in the primary analysis**

We aim here to classify the most common statistical methods used in terms of parametric or non-parametric method. Using the subcategorized defined by (Upton et al, 2008), we want to highlight the most used univariate methods. The following categorization for univariate will include: Student's T-test, ANOVA, ANCOVA, non-parametric test, general linear model, and GLM. We will also highlight how frequent frequentist method are used compared to Bayesian approach.

**Variable extraction**

We extracted variables in the following four categories:

**Publication**: this will include specifically variables related to the presentation of the article: type of the disease; title of the article; first author; year of publication; objective of the article and country.

**Study design**: this includes variables related to the design of the study and related to the statistical methodology such as (1) the sample size, since it can impact the power. Study with small sample size will be subject to bias when estimating the effect, due to the large variance; (2) type of randomization, since it will ensure that the two groups are balanced, (3) the identification of the primary endpoint which can mainly lead to a specific statistical method to use when the primary outcome is a time event, a quantitative or a qualitative (binary) outcome. Other variables are phase of the trial, number of arms or type of blinding.

**Parameters:** Some important parameters giving an overview on the trial duration is follow-up length of the trial and follow-up length for primary endpoint. Other parameters are missing data or dropout rate, since some methods can be more robust when dealing with missing data compared to other. If the participants who drop out are systematically different from those who remain in the study, it can introduce selection bias. This bias may affect the generalizability of the results to the intended population. If those who drop out have experienced adverse effects or lack of efficacy with the treatment, the estimated treatment effect may be biased. Thus, appropriate statistical methods should be chosen to handle missing data and assess the robustness of the results.

**Statistical methodology:** This include primary end point, primary analysis, how estimands are handled, multiplicity interim analysis, significance level, sidedness, and clinical difference. They are ensuring the validity, reliability, and meaningful interpretation of the study results.

Parameter table:

| Variables collected | Value | Description |
|---|---|---|
| Type of disease | Loiasis, mansonellosis, onchocerciasis | Microfilarial infection investigated |
| Title of the article | [Text] | |
| First author | [Text] | |
| Year of publication (registration) | [Integer] | Year of trial publication or registration of the protocol |
| Type of record | publication/registry | |
| Protocol or Statistical Analysis Plan available | yes/no | |
| Primary objective | Efficacy, safety or both | |
| Country | [Text] | Country where study was conducted |
| Number of arms | [Integer] | |
| Intervention | [Text] | Treatment used (excluding control) |
| Doses | [Text] | Dose used for treatment |
| Control | e.g. placebo/standard treatment/ no treatment | Type of control used |
| Trial registration | [Text] | ID of registered trial protocol |
| Method of randomization described | yes/no | Randomization method described in manuscript, protocol, or SAP |
| Type of randomization | e.g., simple randomization, block randomization, stratified randomization | |
| Type of blinding | e.g., double-blind, single-blind, open label | Who was blinded |
| Type of the design | Superiority or non-inferiority | Clinical trial design |
| Sample size calculation | yes or no | Was a sample size calculation provided? |
| Pre-planned sample size | [Integer] | The total number of pre-planned participants |
| Sample size in intervention group | [Integer] | Sample size after randomization (treatment group) |
| Sample size in placebo group | [Integer] | Sample size after randomization (control group) |
| Missing data | [Integer] | Randomized participants lost for primary endpoint data |
| Dropout rate | [Integer] | |
| Follow-up total | [Integer] | |
| Primary endpoint identified | yes or no | Primary endpoint identified in manuscript or protocol |
| Primary endpoint | [Text] | |
| Primary endpoint based on microfilaria | yes/no | |
| Follow-up primary endpoint | [Integer] | |
| Unit | Days, months, years | Unit of time |
| Type of primary outcome | Quantitative/qualitative | |
| Detail of the primary outcome | [Text] | Specification of the primary outcome |
| Summary measure | Arithmetic mean, geometric mean, median, etc. | Summary used for the primary outcome |
| Phase of the RCT | [Integer] | |
| Primary analysis | [Text] | Statistical method for primary outcome |

Continued on next page

| Nr | Title of article/registry | Primary endpoint |
|---|---|---|
| Other statistical methods | [Text] | For secondary analysis |
| Analysis population | ITT or PP | Population used for decision making |
| Statistical method for missing data | [Text] | Approach used |
| Multiplicity (if applicable) | [Text] | Which adjustment for multiplicity was applied? |
| Interim analysis | yes/no | Were interim analyses conducted? |
| Significance alpha | [Integer] | Significance level used |
| Sideness of testing | 1,2 | One-sided or two-sided test |
