# Supplementary material for: Design and analysis of randomized clinical trials for onchocerciasis, loiasis and mansonellosis: A systematic review
Source: PLoS Negl Trop Dis. 2026 Feb 20;20(2):e0013992. doi: 10.1371/journal.pntd.0013992 (PMC12952602; doi:10.1371/journal.pntd.0013992)
Supplement: S2 Text — The PRISMA checklist template was obtained from the PRISMA website (https://www.prisma-statement.org/). (PDF) [file pntd.0013992.s015.pdf]

PRISMA 2020 Checklist

Section and Topic	Item #	Checklist item	Location where item is reported
**TITLE**			
Title	1	Identify the report as a systematic review.<br>Design and analysis of randomized clinical trials for onchocerciasis, loiasis and mansonellosis: A systematic review	Page 1
**ABSTRACT**			
Abstract	2	See the PRISMA 2020 for Abstracts checklist.	Page 1
**INTRODUCTION**			
Rationale	3	Describe the rationale for the review in the context of existing knowledge.<br>To support planning for the eWHORM study, we conducted a systematic review of RCTs in onchocerciasis, loiasis, and mansonellosis, focusing on their trial design.	Page 3, line 91-93
Objectives	4	Provide an explicit statement of the objective(s) or question(s) the review addresses.<br>Our primary aim was to provide a comprehensive overview of the statistical methods used, with emphasis on qualitative or quantitative primary endpoints. Second, we applied the estimand framework introduced in the International Council for Harmonisation guideline, ICH E9(R1) addendum [29], which provides a systematic approach to defining treatment effects in clinical trials, with explicit consideration of intercurrent events such as treatment discontinuation, use of rescue medication, or death. We extracted information on the estimand attributes population, variable (endpoint), summary measure, as well as the strategies to deal with intercurrent events. Additionally, we described the trials with respect to sample size and number of arms, and summarized key statistical parameters relevant for study design, including advanced design features such as interim analyses and multiplicity adjustment.	Page 3, line 93-103
**METHODS**			
Eligibility criteria	5	Specify the inclusion and exclusion criteria for the review and how studies were grouped for the syntheses.<br>The review addressed RCTs assessing efficacy and/or safety in onchocerciasis, loiasis, and mansonellosis, identified from articles in peer-reviewed journals or registered in the clinical trial registries between January 1, 2000 and December 31, 2024. For trials published as an article, this time frame refers to the publication date. If a trial had not yet been published and only a record in a registry was identified, the time frame refers to the registration date in the registry. We excluded studies focusing solely on new methods, short reports, RCTs in other diseases, and Phase I RCTs, and trials assessing pharmacokinetics.	Page 4, line 117-124
Information sources	6	Specify all databases, registers, websites, organisations, reference lists and other sources searched or consulted to identify studies. Specify the date when each source was last searched or consulted.<br>The search was conducted in the literature databases PubMed (https://pubmed.ncbi.nlm.nih.gov/) and the four registry databases ClinicalTrials.gov (https://ClinicalTrials.gov/), WHO International Clinical Trials Registry (ICTR) (https://trialsearch.who.int/), International Standard Randomised Controlled Trial Number (ISRCTN) (https://isrctn.com/) registry, and the Pan African Clinical Trials Registry (PACTR) (https://pactr.samrc.ac.za/). The search was limited to RCTs in humans from January 1, 2000 to December 31, 2024 in English or French. The final query in the databases was carried out on January 23, 2025. The search terms used in the individual databases are specified in S1 Table.	Page 4, line 107-114
Search strategy	7	Present the full search strategies for all databases, registers and websites, including any filters and limits used.<br>Pubmed:<br>(”loiasis”[MeSH Terms] OR ”loiasis”[All Fields] OR ”Loa loa”[All Fields]) AND ((randomizedcontrolledtrial[Filter]) AND (2000:2024[pdat]))<br>(”mansonella”[MeSH Terms] OR ”mansonella”[All Fields] OR ”mansonelliasis”[MeSH Terms] OR ”mansonelliasis”[All Fields] OR<br>”mansonelliases”[All Fields]) AND ((randomizedcontrolledtrial[Filter]) AND (2000:2024[pdat]))<br>”onchocerciasis”[MeSH Terms] OR ”onchocerciasis”[All Fields] OR ”onchocerciases”[All Fields] OR ”onchocerciasis”[MeSH Terms] OR<br>”onchocerciasis”[All Fields]) AND ((randomizedcontrolledtrial[Filter]) AND (2000:2024[pdat]))	S1 Table

Section and Topic	Item #	Checklist item	Location where item is reported
		Other registries:<br>Loiasis OR Loa Loa OR Loiases<br>mansonella OR mansonelliasis OR mansonelliases<br>onchocerciasis OR onchocerciases OR onchocerciasis	
Selection process	8	Specify the methods used to decide whether a study met the inclusion criteria of the review, including how many reviewers screened each record and each report retrieved, whether they worked independently, and if applicable, details of automation tools used in the process.<br>All articles or records identified by the search were initially screened based on the title and the abstract (if available) to determine eligibility for the specific disease. In the next step, the full text or the information from the registry was screened for further exclusion. Duplicates of trials identified in PubMed and in a registry were identified via the registry number. Records identified in WHO ICTR were all either duplicated in ClinicalTrials.gov, ISRCTN, or PACTR and linked via the same registry number. After the identification of duplicates, the final set of included trials was determined. The selected articles and/or records were screened and the relevant information was collected in a spreadsheet. For some trials the study protocol was also available from the registry or provided as supplementary material in the article and additional information from the study protocol was retrieved. If the study protocol and article had different information, the information was retrieved from the article. The first author conducted the initial search, applied the exclusion criteria, and screened the identified articles. One additional reviewer performed an independent assessment. Consistency between independent reviewers was evaluated by consensus review to resolve any discrepancies.	Page 4, line 126-140
Data collection process	9	Specify the methods used to collect data from reports, including how many reviewers collected data from each report, whether they worked independently, any processes for obtaining or confirming data from study investigators, and if applicable, details of automation tools used in the process.<br>The selected articles and/or records were screened and the relevant information was collected in a spreadsheet. For some trials the study protocol was also available from the registry or provided as supplementary material in the article and additional information from the study protocol was retrieved. If the study protocol and article had different information, the information was retrieved from the article. The first author conducted the initial search, applied the exclusion criteria, and screened the identified articles. One additional reviewer performed an independent assessment. Consistency between independent reviewers was evaluated by consensus review to resolve any discrepancies.	Page 4, line 132-140
Data items	10a	List and define all outcomes for which data were sought. Specify whether all results that were compatible with each outcome domain in each study were sought (e.g. for all measures, time points, analyses), and if not, the methods used to decide which results to collect.<br>Outcomes: See S2 Table<br>To extract information from the trials we applied the estimand framework as introduced in the ICH E9(R1) addendum [29] focusing on the description of the attributes population, variable (endpoint), strategies for handling intercurrent events, and summary measure. Although only a small number of the selected trials explicitly referenced the estimand framework, the articles, registries and/or study protocols were systematically examined to identify and extract these attributes. As described in the estimand framework, population was described by the disease and specific inclusion and exclusion criteria (lower and upper limits for mf, age, and weight). For the intercurrent events, we inferred the estimand strategy based on the analysis population used - intention-to-treat (ITT) or per protocol (PP) - when explicitly stated. If the specification of ITT or PP could not be extracted (by the corresponding words), it was recorded as missing. We did not extract specific intercurrent events such as death, treatment discontinuation or compliance. The summary measure describes how outcomes were aggregated and compared between treatment groups in the primary analysis. This included statistics such as arithmetic or geometric means, medians or risk differences. As this was inconsistently reported across studies, we provide an overview of applied descriptive statistics. The attribute variable refers to the primary endpoint and it was categorized in each trial as a qualitative or quantitative variable based on either follow-up values or changes from baseline to follow-up. We also recorded the duration of follow-up used for the primary endpoint. The treatment attribute includes<br>types of intervention(s) and control(s). In addition to the estimand-related data, we extracted general trial characteristics such as article title, first author, publication/registration year, study objective, registry number, number of arms, country, planned/calculated and actual randomized	S2-S4 Table, line 142-185

Section and Topic	Item #	Checklist item	Location where item is reported
		sample sizes, randomization method, percentage of missing data for the primary analysis, handling of missing data, latest follow-up, the statistical methods applied for the primary endpoints, implementation of interim analyses, and multiplicity control. The full list can be found in S2 Table.<br>The planned sample size was determined either from the sample size calculation provided in the publication, the study protocol or the reported enrollment number listed in the registry. The actual sample size refers to the number of participants randomized. Again, information was only extracted if the information was explicitly reported. For randomization methods, the information was recorded as unclear if randomization was mentioned in the title or abstract but the method of randomization was not specified.<br>For trials reporting multiple follow-up time points for the primary endpoint, we recorded the maximum follow-up duration. In general, information on attributes or other study parameters was only extracted when explicitly specified in the publication, the registry and/or the study protocol. Otherwise the parameter was recorded as missing.<br>A protocol of the systematic review is available as supporting information S3. The review was not registered. Excel files (i) of all identified articles and/or records in registries with reasons for exclusion are provided in S4 Table and (ii) of all 44 included trials and the extracted information in S5 Table. The Excel files are also available at: https://gitlab.com/mougeni/ewhorm scientificreview	
	10b	List and define all other variables for which data were sought (e.g. participant and intervention characteristics, funding sources). Describe any assumptions made about any missing or unclear information.<br>See Table S2<br>For the intercurrent events, we inferred the estimand strategy based on the analysis population used - intention-to-treat (ITT) or per protocol (PP) - when explicitly stated. If the specification of ITT or PP could not be extracted (by the corresponding words), it was recorded as missing.<br>The planned sample size was determined either from the sample size calculation provided in the publication, the study protocol or the reported enrollment number listed in the registry. The actual sample size refers to the number of participants randomized. Again, information was only extracted if the information was explicitly reported. For randomization methods, the information was recorded as unclear if randomization was mentioned in the title or abstract but the method of randomization was not specified.<br>For trials reporting multiple follow-up time points for the primary endpoint, we recorded the maximum follow-up duration. In general, information on attributes or other study parameters was only extracted when explicitly specified in the publication, the registry and/or the study protocol. Otherwise the parameter was recorded as missing.	S2 Table, line 149-153<br>170-180
Study risk of bias assessment	11	Specify the methods used to assess risk of bias in the included studies, including details of the tool(s) used, how many reviewers assessed each study and whether they worked independently, and if applicable, details of automation tools used in the process.<br>The first author conducted the initial search, applied the exclusion criteria, and screened the identified articles. One additional reviewer performed an independent assessment. Consistency between independent reviewers was evaluated by consensus review to resolve any discrepancies.<br>In general, information on attributes or other study parameters was only extracted when explicitly specified in the publication, the registry and/or the study protocol. Otherwise we considered the parameter as missing	Line 137-140, 178-180
Effect measures	12	Specify for each outcome the effect measure(s) (e.g. risk ratio, mean difference) used in the synthesis or presentation of results.<br>Main characteristics were shown for each trial (registration number, primary objective, phase, type of blinding, number of arms, whether a sample size calculation was reported, actual sample size, analysis population, title of article/registry, primary endpoint, intervention and control details). Study characteristics were summarized using descriptive statistics, mainly absolute and relative frequencies of characteristics were computed for each disease and across diseases and for the time periods 2000-2012 and 2013-2024. For planned sample sizes and number of arms, medians and interquartile ranges (IQR) were reported. Actual sample sizes of participants randomized were depicted by boxplots for control and intervention groups per disease, and further stratified by type of control group (placebo/no treatment versus standard drug/dose). In trials with more than one intervention group, average sample sizes were shown. Additional boxplots summarized follow-up duration (overall and for the primary endpoint) and percentage of missing values by disease. Barplots were used to show the frequency of statistical methods applied	Line 187-202

Section and Topic	Item #	Checklist item	Location where item is reported
		to quantitative and qualitative endpoints overall and for time periods 2000-2012 and 2013-2024. R version 4.3.1 (R Foundation for Statistical Computing, Vienna, Austria) was used for all statistical analyses and plots.	
Synthesis methods	13a	Describe the processes used to decide which studies were eligible for each synthesis (e.g. tabulating the study intervention characteristics and comparing against the planned groups for each synthesis (item #5)).<br>Not applicable	
	13b	Describe any methods required to prepare the data for presentation or synthesis, such as handling of missing summary statistics, or data conversions.<br>Not applicable	
	13c	Describe any methods used to tabulate or visually display results of individual studies and syntheses.<br>Not applicable	
	13d	Describe any methods used to synthesize results and provide a rationale for the choice(s). If meta-analysis was performed, describe the model(s), method(s) to identify the presence and extent of statistical heterogeneity, and software package(s) used.<br>Not applicable	
	13e	Describe any methods used to explore possible causes of heterogeneity among study results (e.g. subgroup analysis, meta-regression).<br>Not applicable	
	13f	Describe any sensitivity analyses conducted to assess robustness of the synthesized results.<br>Not applicable	
Reporting bias assessment	14	Describe any methods used to assess risk of bias due to missing results in a synthesis (arising from reporting biases).<br>Not applicable	
Certainty assessment	15	Describe any methods used to assess certainty (or confidence) in the body of evidence for an outcome.<br>Not applicable	
**RESULTS**			
Study selection	16a	Describe the results of the search and selection process, from the number of records identified in the search to the number of studies included in the review, ideally using a flow diagram.<br>We identified 268 articles and/or records in registries (Fig 1). Overall, 122 reports were removed as duplicates. From the 146 remaining reports, 56 records were removed based on the abstract or title. Furthermore, 46 reports were removed from the remaining set of 90 studies for not meeting the inclusion criteria (see S4 Table). The remaining 44 trials were included in the systematic review and the full text was assessed (23 (52%) for onchocerciasis, 16 (36%) for loiasis, and 5 (11%) RCTs for mansonellosis).	Line 204-209, Figure 1, S4 Table
	16b	Cite studies that might appear to meet the inclusion criteria, but which were excluded, and explain why they were excluded.<br>Not applicable	
Study characteristics	17	Cite each included study and present its characteristics.<br>Table 1 summarizes the key characteristics of the 44 identified trials, S6 Table lists the titles of the articles/registries and the primary endpoints (all extracted information can be found in S5 Table). For some trials, information was incomplete. For 18 (41%) of trials no publication was available, and several parameters were missing such as study phase, whether a sample size calculation was performed (sample size calculation), actual sample sizes of participants randomized, or analysis population. Among these 18 trials, 3 provided a study protocol or a statistical analysis plan (SAP) and one master protocol was available for the 3 eWHORM trials. In 10 trials with a published manuscript, no registration number was found in the full text. Matching these articles with registry entries was limited due to inconsistencies in registry	Tables 1 and 2, S5 Table<br>Lines 214-227

Section and Topic	Item #	Checklist item	Location where item is reported
		identifiers, as the intervention name or study design alone often led to ambiguity or potential mismatches. Thus, only records with the same registry number were matched. For 16 trials (36%) both the published manuscript and a registry number were available (note that for earlier trials registration was not mandatory).	
Risk of bias in studies	18	Present assessments of risk of bias for each included study.<br><br>Not applicable	
Results of individual studies	19	For all outcomes, present, for each study: (a) summary statistics for each group (where appropriate) and (b) an effect estimate and its precision (e.g. confidence/credible interval), ideally using structured tables or plots.<br><br>Ad a)<br><br>All trials tested a superiority hypothesis. A planned sample size was reported in 36 trials (82%) derived either from the sample size calculation presented in the article or the target sample size reported in the registry. In these trials, the median total number of planned participants was 166 (IQR: 82-240). However, only 23 trials (52%) including 15 with a published manuscript reported a formal sample size calculation (for 6 trials this information was obtained from the authors). For loiasis, smaller total planned sample sizes were observed (median 99, IQR: 60-160) compared to the other diseases (mansonellosis: median 180, IQR: 105-200, onchocerciasis: median 220, IQR: 153-323). Fig 3 shows the actual sample sizes per arm for the control and intervention groups for the primary endpoint, stratified by disease. In trials with multiple intervention groups, average sample sizes are shown. Most trials used a 1:1 allocation ratio as shown by the horizontal lines crossing the boxes for each disease. Information on trial phase was available for 26 trials. Reported phases were Phase 2 (19 trials), Phase 2/3 (1 trial), Phase 3 (5 trials), and Phase 4 (1 trial). For some trials, the registries listed the phase as ”not applicable”. Regarding the analysis population for the primary analysis, 7 trials used PP and 9 used ITT analysis. In total, 64% of the trials did not specify the type of analysis population (see Table 1). Twenty-two trials (50%) reported inclusion criteria based on mf counts. Among these, 9 trials specified both lower and upper mf thresholds. For loiasis, 75% of trials reported mf-based inclusion criteria, whereas the corresponding proportions were lower for onchocerciasis (39%) and mansonellosis (20%). Detailed lower and upper bounds for mf for each trial as well as bounds for age and weight are shown in S7 Table<br><br>**Interventions** In the 44 trials, different interventions were evaluated, including IVM, ALB, levamisole, DEC, DEC-medicated salt, moxidectin, a combination of quinine, chloroquine, amodiaquine, and artesunate, emodepside, reslizumab, rifampin, azithromycin, DOXY and others (for details on each study, see S8 Table). The number of study arms, including the control group, varied by disease: loiasis and mansonellosis trials typically had two or three arms (median 2 for mansonellosis and 3 for loiasis), while onchocerciasis trials often had more than three arms (median: 4, IQR: 3-4). Placebo was the most frequent control in loiasis (10/16) but not in onchocerciasis studies, where a standard drug/dose was most frequently reported as control (15/23 trials). For mansonellosis, placebo and standard drug/dose were each reported twice (see Table 2). Control groups receiving ”no treatment” were reported in two trials in the earlier time period (2000-2012), but were not observed in the later period (2013-2024) (see S9 Table).<br><br>**Randomization and blinding** Among the 44 trials, 24 (55%) reported details on the randomization procedure (Table 2). Stratified randomization was the most frequently reported method (14 trials, 32%), followed by block randomization (7 trials). Note that all trials with block randomization were from time period 2013-2024 (S9 Table). Stratification was commonly based on mf load, other stratification factors included age, sex, and mf load, or status of mf [1, 27, 35]. Double-blind designs predominated (25/44, 57%) (see Table 1). Open-label trials accounted for nearly one-third of studies (14/44, 32%), whereas single-blind and assessor-blind designs were rare (each 4/44, 9%). Blinding was unspecified in one trial (2.3%). By disease, double-blinding was most frequent in loiasis trials (11/16, 69%), followed by mansonellosis (3/5, 60%) and onchocerciasis (11/23, 48%). Open-label designs were particularly common in onchocerciasis (10/23, 43%) and mansonellosis (2/5, 40%), but less frequent in loiasis (2/16, 13%)<br><br>**Primary endpoints** The primary objective was defined for 37 trials (see Table 1), either in the methods sections of the manuscripts or the	Lines 229-345,<br><br>Tables 1-4

Section and Topic	Item #	Checklist item	Location where item is reported
		registries. Of these, 33 trials had a single primary objective, which was related to efficacy in 27 and to safety in 6 trials. In further 4 trials, both efficacy and safety were defined as the primary objective. In the remaining 7 trials, the definition of the primary endpoint was missing. Further details on the specification of the primary endpoint can be found in S6 Table. Among the 31 trials with a primary efficacy endpoint, 14 trials defined a quantitative endpoint, 15 a qualitative endpoint, one trial reported both (estimand attribute variable/endpoint), and one was not clearly defined (Table 3). Qualitative endpoints were reported more frequently than quantitative endpoints for onchocerciasis trials only. In 17 trials, the primary efficacy endpoint was defined based on measurement of mf values. Of these, 6 trials assessed mf qualitatively, for example by evaluating whether participants reached a pre-specified threshold or fell below a defined mf level. Ten trials assessed mf quantitatively as primary endpoint and one trial used both. Among the trials with a quantitative primary endpoint, five trials focused on the changes in mf from baseline to follow-up, whereas 6 assessed mf values at follow-up (results comparing the time periods 2000-2012 and 2013-2024 can be found in S10 Table). Fig 4 shows boxplots of follow-up times for the primary endpoints and the complete follow-up of the trials, along with the percentage of missing data at the primary endpoint time point. The follow-up durations (both for complete and primary endpoint) were generally shortest for trials investigating loiasis.<br><br>**Statistical methods**<br><br>**Statistical methods for quantitative endpoints** The most commonly reported inferential methods for quantitative efficacy or safety primary endpoints were univariable analyses (≥ 70%), whereas multivariable methods were only used in approximately 25% of the trials. For quantitative primary endpoints, the Mann-Whitney U or Kruskal-Wallis tests (MWKW) were the most frequently applied methods (12 trials), followed by linear mixed models (LMM) (see Fig 5 and S11 Figure, stratified by time period). Two other trials used a generalized linear mixed model (GLMM) for quantitative endpoints of either a secondary (efficacy) endpoint or an endpoint which was neither primary or secondary. In these models, random effects included study participant (to adjust for multiple worms per person) or study site. Fixed effects included baseline mf, level of infection, sex, treatment, and treatment by covariate interactions. Results of the analyses for the quantitative primary endpoints were reported by several summary measures within studies, such as geometric and arithmetic mean and median. However, summary measures and analysis methods were often not aligned. A primary summary measure was only defined in 11 trials (geometric mean in 6, median in 2, and concordance in 3 trials). The concordance (also called probabilistic index or relative effect [64]) is a summary measure for the Mann-Whitney U test and was defined as summary measure in the eWHORM substudies [49]<br><br>**Statistical methods for qualitative endpoints** For trials with primary qualitative endpoints, the chi-squared or Fisher's exact tests were the most commonly used methods (7 trials), 4 trials reported logistic regression and 1 generalized linear mixed model(see Fig 5). The summary measure proportion for the analyses was explicitly defined in 4 trials.<br><br>**Multiplicity adjustment** Although 30 trials had more than two arms, only six reported methods to control the familywise error rate (FWER): Two trials applied the Bonferroni correction [24, 53]. One trial used a closed testing procedure to control the FWER which involved a global test of equal rates of fertile female worms for four treatments and all intersection hypotheses considering three treatments at level α, followed by pairwise intersection hypotheses tested at level α/2 [2]. In the three eWHORM substudies a partial conditional error approach was embedded within a closed testing procedure to ensure strong control of the FWER for the multiple doses and the two stages [49]. One trial planned to conduct an interim analysis after all patients had been recruited and had reached their 3 month follow-up. Based on these interim data, the total length of follow-up was planned to be re-evaluated [58]<br><br>**Handling of missing data** Several trials reported approaches to handle missing data for the primary endpoint. One trial applied the "extreme analysis" to assess the significant effect of the intervention evaluating the robustness of the treatment effect under a worst-case assumption [17]. Other approaches were linear interpolation to impute the missing values [25], a log-rank test to compare the drop-out patterns between the groups over time [33], assumption of a worst-case scenario [35], and multiple imputation using logistic regression methods [42]. In [47] sensitivity analyses for the primary efficacy endpoint were proposed to explore the impact of various imputation algorithms on the primary efficacy endpoint.	

PRISMA 2020 Checklist

Section and Topic	Item #	Checklist item	Location where item is reported
		In the eWHORM substudies missing data were planned to be handled by multiple imputation methods specifically tailored for zero-inflated data [65].<br><br>Ad b) Not applicable	
Results of syntheses	20a	For each synthesis, briefly summarise the characteristics and risk of bias among contributing studies.<br>Not applicable	
	20b	Present results of all statistical syntheses conducted. If meta-analysis was done, present for each the summary estimate and its precision (e.g. confidence/credible interval) and measures of statistical heterogeneity. If comparing groups, describe the direction of the effect.<br>Not applicable	
	20c	Present results of all investigations of possible causes of heterogeneity among study results.<br>Not applicable	
	20d	Present results of all sensitivity analyses conducted to assess the robustness of the synthesized results.<br>Not applicable	
Reporting biases	21	Present assessments of risk of bias due to missing results (arising from reporting biases) for each synthesis assessed.<br>Not applicable	
Certainty of evidence	22	Present assessments of certainty (or confidence) in the body of evidence for each outcome assessed.<br>Not applicable	
**DISCUSSION**			
Discussion	23a	Provide a general interpretation of the results in the context of other evidence.	
	23b	Discuss any limitations of the evidence included in the review.<br>This review was limited by sparse or incomplete reporting for several trials indicating a need for more comprehensive and transparent documentation. A clearer definition of study objectives and analytical strategies could be achieved by adopting the estimand framework outlined in the ICH E9 (R1) addendum on estimands and sensitivity analysis in clinical trials [29] in addition to compliance with the CONSORT<br>statement for reporting RCTs [29, 100]. ICH E9(R1) provides a framework to align the design and analysis of a clinical trial to the trial objective, whereas the CONSORT statement provides guidance for the transparent and standardized reporting of RCTs. Among the reviewed trials, only the eWHORM trial explicitly applied the estimand framework. For the remaining trials, we retrospectively retrieved information on the five estimand attributes population, treatment, variable (endpoint), intercurrent events and summary measure, wherever possible. The absence of reported information for a given attribute does not necessarily imply that it was not defined in the trial documents, but rather that it was not included in the sources we reviewed.	Lines 600-612
	23c	Discuss any limitations of the review processes used.<br>Not applicable	
	23d	Discuss implications of the results for practice, policy, and future research.<br>This systematic review identified both strengths and weaknesses in the design, analysis and reporting of RCTs for onchocerciasis, loiasis, and mansonellosis. Several trials were well planned and comprehensive information was available either in published manuscripts or trial registries. However, for several trials, the reported information was sparse, inconsistent, or incomplete. This underscores the need for standardized, structured, and transparent reporting. Adopting established frameworks such as CONSORT and ICH E9 (R1) estimand approach would improve transparency and strengthen alignment between trial objectives, analyses, and reported conclusions.	Lines 625-646

PRISMA 2020 Checklist

Section and Topic	Item #	Checklist item	Location where item is reported
		A major challenge in trials on onchocerciasis, loiasis, and mansonellosis is the typically small sample size, which increases the risk of baseline imbalance between treatment groups. The use of block and stratified randomization when key prognostic factors are identified can improve balance on important covariates. Proper blinding can reduce bias by helping to avoid systematic differences in care or outcome assessment between arms. In addition, future trials should consider statistical methods aligned to zero-inflated or skewed endpoints, adjustment for covariates, clear definition of the primary summary measure, appropriate handling of missing data, and adjustment for multiplicity when multiple hypotheses are tested. Finally, adaptive designs can be considered as an option during planning, allowing prespecified design modifications, for example early stopping, sample size re-estimation, or dropping or adding of treatment arms. Whether an adaptive design should be chosen depends on trial objectives as well as practical constraints and should be evaluated on a case-by-case basis.	
**OTHER INFORMATION**			
Registration and protocol	24a	Provide registration information for the review, including register name and registration number, or state that the review was not registered.<br>The review was not registered.	Line 181-182
	24b	Indicate where the review protocol can be accessed, or state that a protocol was not prepared.<br>A protocol of the systematic review is available as supporting information S3,	Line 181
	24c	Describe and explain any amendments to information provided at registration or in the protocol.<br>Not applicable	Lines 125-128
Support	25	Describe sources of financial or non-financial support for the review, and the role of the funders or sponsors in the review.<br>This work is part of the EU project eWHORM: The eWHORM project (No 101103053) supported by the Global Health EDCTP3 Joint Undertaking and its members as well as the Swiss Confederation. Views and opinions expressed are those of the author(s) only and do not necessarily reflect those of the European Union or the Global Health European and Developing Countries Clinical Trials Partnership (EDCTP3). Neither the European Union nor the granting authority can be held responsible for them.<br>MBR is a Serra H´unter Fellow and was additionally supported by Grant PID2023-148033OB-C21 funded by MICIU/AEI/10.13039/501100011033 and by FEDER/UE.<br>MPH is funded under Germany's Excellence Strategy—EXC2151-390873048 and is members of the German Center for Infection Research (DZIF). MPH received funding from the German Center for Infection Research (TTU 09.701).	Lines 719-739
Competing interests	26	Declare any competing interests of review authors.<br>None	
Availability of data, code and other materials	27	Report which of the following are publicly available and where they can be found: template data collection forms; data extracted from included studies; data used for all analyses; analytic code; any other materials used in the review.<br>Not available	

From: Page MJ, McKenzie JE, Bossuyt PM, Boutron I, Hoffmann TC, Mulrow CD, et al. The PRISMA 2020 statement: an updated guideline for reporting systematic reviews. BMJ 2021;372:n71. doi: 10.1136/bmj.n71.
This work is licensed under CC BY 4.0. To view a copy of this license, visit https://creativecommons.org/licenses/by/4.0/
